# How do recommender systems learn political opinions? A semi-synthetic step-by-step experiment

**DOI:** 10.1371/journal.pone.0349341

**Published:** 2026-05-26

**Authors:** Tim Faverjon, Jean-Philippe Cointet, Pedro Ramaciotti

**Affiliations:** 1 médialab Sciences Po, Paris, France; 2 Learning Planet Institute, Learning Transitions, CY Cergy Paris University, Paris, France; 3 Complex Systems Institute of Paris Ile-de-France ISC-PIF CNRS, Paris, France; Hunan Normal University, CHINA

## Abstract

Recommendations play a crucial role in shaping informational diets on social media, raising concerns regarding potential consequences such as political segregation. We take an algorithm explanability approach, as opposed to a description of recommendations, to show how recommenders inadvertently create geometrical representations of the ideological position of users with minimal and ubiquitous platform data, and how this impacts content diets. In comparison to work showing the existence of ideological structures in machine representations, we provide a step-by-step causal explanation of their formation. To achieve this, we compute synthetic recommendations with a model trained on real-world data from a panel of nearly 40 thousand X users and the contents they shared on the platform. We show that elementary recommendation principles trained on content dissemination data produce a spatial representation of the Left-Right positions of users in our panel in the recommender, which is also independent of other common attributes, such as age and gender. We explore the consequences of our findings by modifying these ideological representations in the recommender and analyzing the trade-off in resulting recommendations in terms of political leaning, diversity, and relevance of offered contents.

## Introduction

Social platforms play an increasingly central role in the dissemination of information among the public, including political content and discourse [1]. The impact that social media may have more broadly in society has thus raised concerns related to the diversity of content shown online [2], the effects on public opinion and polarization [3], and informational segregation [4]. The study of these phenomena on social media has now become an established field of scientific research.

One of the key elements of social media and online systems, among those that raise concern, are recommendation algorithms, or recommender systems. Because of the abundance of informational resources, these systems have become ubiquitous and indispensable, and drive most of the consumption of content in social media [5]. Recommender systems in social media have also become central to user satisfaction and drive the business model of platforms [6,7]. Some of the concerns regarding the potential impacts of recommender systems include the possibility that they peddle radicalizing content [8], or that, by providing only more of what users already like, they can reduce their informational horizons, with potential negative consequences for the broader public sphere and national politics [9–11]. This potential narrowing effect on informational horizons, for instance, has raised specific concerns regarding political information [12,13]. While individuals with different political preferences will naturally be exposed to information from political actors in uneven ways (e.g., even before the invention of the internet), the specific concern regarding recommender systems relates to the degree to which this phenomenon may manifest [14–16]. It has also been shown that algorithmic recommendations may, in some specific cases, on the contrary, increase the diversity of content [17,18]. Both the effects of these phenomena of concern and their prevalence constitutes in itself a challenging object of research counting numerous research works [19,20]. These potential effects are further compounded by feedback dynamics, whereby internally held opinions shape the behavioural trace data that users produce, which in turn inform the algorithms governing content exposure — and may thereby influence opinions themselves. Our work contributes to the understanding of how internally held political opinions, and their associated behavioural traces, shape algorithmic content curation.

**Algorithm transparency: auditing vs explainability.** To address these issues, numerous studies advocate and develop methods for increasing transparency of recommendation algorithms [21–23], with a particular emphasis on showing how they process sensitive data, such as political opinions [24]. Among different existing methods or frameworks for operationalizing transparency of algorithms, two approaches have accumulated a specially large number of works, tools, and interest from practitioners, regulators, and policy makers: algorithm *auditing* and *explainability*.

Algorithm auditing involves the systematic assessment of the outcomes of recommendations, i.e., what was recommended to whom. By characterizing users and recommended items (e.g., posts, videos, contents, and even recommended friends), auditing involves measuring properties of recommendations such as biases with respect to baselines [15], diversity [25], novelty [26], or directly their effects on online social systems [27]. Algorithm explainability, on the other hand, focuses on producing human-understandable explanations of the computational processes determining a recommendation, allowing an examiner to understand the steps and the factors that explain why an item is recommended over another to a given user [28]. By understanding the mechanisms underlying recommendations, undesired outcomes can be identified and measured, potentially enabling algorithm design principles to avoid them. Algorithm explainability is notably challenging because of the complexity of some of these systems, a difficulty encapsulated by the coined phrase *black box*, used to refer to some (recommendation) algorithms.

**AI explainability and online politics.** In the context of studies addressing political phenomena online (e.g., polarization or informational segregation along ideological lines), these challenges are compounded by the need of leveraging frameworks of analysis for relevant constructs, typically including those of political attitudes (positive or negative dispositions towards political issues) or ideologies (collections or structures of attitudes that are highly correlated). Most studies on social media and algorithms in political settings focus on the most relevant ideological divides, such as the Left-Right or the Liberal-Conservative ideological divide. Algorithm explainability in this context takes interest in questions such as: How (and to what degree) does the perceived ideological stance of an item *i* (e.g., a news article) and that of the user *u* receiving the recommendation participate and affect the computation of the recommendation? Notably, the involvement of these constructs in research design and experiments is challenging also because of the difficulty in measuring and linking the ideology or issue positions of users and those of content by using digital trace data. While self-positioning surveys administered to panels of users remain the traditional means of measuring ideology positions for individuals, it is still challenging to match these responses to users and the recommendations they are exposed to, let alone the data they generate and that determines the recommendations they see [29].

In addition to these challenges, scholars have also noted that the traditional single-dimensional Left-Right or Liberal-Conservative frame of ideological analysis, while well-suited for case studies in the US, might prove insufficient for more general cases. Indeed, a long tradition in compared politics studies the dimensionality of politics in different national settings [30], showing that, in most countries, additional ideology or issue dimensions are needed to account for relevant choice data: from how individuals vote [31], to how they produce data in social media [32].

**AI explainability and recommender systems.** Explaining the role of ideology or issue positions in computations involves first establishing these attributes for relevant users and contents, to then investigate if and how they participate in computations (even inadvertently: where they are not part of the design of the algorithm nor are they part of the input as data). Various and diverse forms of algorithmic explanation exist [28]. In this work, we will focus on explainability methods that aim to identify the impact of certain implicit features or attributes, namely the political ideological and issue positions of users on the model learned by the recommender and leveraged in computation of recommendations (commonly called attribution method [33]). We emphasize that the study of these attributes considers them to be *implicit* in the sense that no input variable related to politics is available to the system during training or during exploitation. Instead, we seek to understand the mechanisms through which a recommender system is capable of independently creating these attributes as part of the abstract model within its so-called black box. Furthermore, our objective is not to explain the structure of the model before training (e.g., describe its architecture) but rather to understand the model’s functioning once trained, presenting a *post hoc* explanation method [34].

Regarding the practical setting, this work focuses on user-content recommendation algorithms, which aim to predict which content will be interesting or relevant to a given user. Recommenders of this kind (but also of other kinds), in the current state of the art, work mainly in two steps [35]. The first step consists of estimating, from the available data traces (e.g., how has each user interacted with past content) a model. In many real cases, and in the case we propose in this article, this is a spatial model in which users and items have positions, i.e., an embedding, which encodes information relevant from the used data traces during training. The second step (usually computationally less intensive than the first) estimates, from this embedding, the numerical scores or indexes representing the degree to which an item is estimated to be relevant for a user. Understanding the hidden semantics of these embedding spaces (e.g., which user or item construct or attribute relates to which dimension of the space), while complex, generally provides the opportunity of proposing mechanistic explanations to recommendations. This is why several methods for explaining recommendation algorithms rely on interpreting the dimensions of embedding spaces [36].

**Our case study.** In this article, we propose a semi-synthetic experiment to show and explain in detail, step-by-step, how recommender systems infer implicit ideological stances for users and contents, with this goal being encoded in the design, and only by using trace data during training. By semi-synthetic, we mean an experimental design that combines real-world observational data (i.e., actual engagement traces of real Twitter users with estimated ideology positions) with a synthetic recommendation model that we train ourselves. This hybrid approach allows us to work in a realistic setting while retaining full access to the internals of the trained model, which would otherwise be inaccessible. We select an ubiquitous recommender system engine that is at the core real-world recommenders that have been deployed in the tech industry: matrix factorization (used, e.g., for Netflix videos [6], or Twitter ads [37]). Matrix factorization recommender systems use training trace data to infer abstract latent positions of users and items in some embedding space, and that results in relevance scores computed as the inner product between these positions for user-content pairs. Our experiment is semi-synthetic: we begin with real-world data on how users with known ideological leanings engage with news content on X/Twitter (Twitter at the time of our collection, thus hereinafter just Twitter), but we compute recommendations by training a model ourselves, without knowledge or *real* recommendations that might have been proposed by the platform to the real users that we consider. This combination of real-world data and synthetic recommendations allows us to instantiate our experiment in realistic settings, while at the same time overcoming one of the major hurdles of platform research, i.e., data accessibility: while we do have access to user engagement data via the platform’s API, it does not offer data on which items were recommended to whom, neither any data on the trained model used by the platform. Training our own algorithm also accomplishes then the crucial objective of rendering the trained model accessible for analysis leading to our main research question: Is information on the ideological leaning of users or contents encoded in the embedding space without it being specified as input data nor as an objective of the training operation? With this empirical research design, we seek to provide the first fully mechanistic, step-by-step explanation of how algorithms learn ideological leanings of users. A central part of our analysis is the comparison between the learned representations or embeddings computed by the algorithm during training on the one hand, and externally or independently developed attributes or features for users and contents, on the other. We leverage a dataset of 40.000 Twitter users with estimated ideological positions on the Left-Right scale and on a scale measuring attitudes towards elites and institutions, related to populism, a leading emerging dimension in several countries. We also include in our analysis covariates such as demographic and occupational groups. The possibility of analyzing ideological features in learned representations achieved by recommender systems using these were was preliminary suggested by Faverjon et al. [38]. In this study we build on this study to conduct the first explanatory analysis with quantitative measurements of learned ideological features.

**Relevance.** We show that trivial data traces (i.e., user engagement with news content) available to most recommenders in the most used platforms, and ubiquitous and simple algorithmic recommendation procedures such as matrix factorization, which are key to several real-world applications, are enough for a recommender to independently develop an internal dimension that characterizes users and content by ideology. We achieve this by overcome one of the main hurdles in the study of political dynamics online, matching both users and news content items with political ideology descriptions, and by deploying this dual semi-synthetic and step-by-step strategy. Acknowledging the richness of AI explainability methods in the literature (a survey of such methods falls outside our scope), our own explainability metrics are resolutely adapted to our case study. In contrast, our explanation methods highlight, for the type of recommendation algorithm we use, the geometrical features of encoded information. Our results are relevant for at least two reasons. First, it opens the possibility of producing explainability tools relating to political variables, and thus the possibility of addressing concerns related to political phenomena such as polarization or informational segregation directly by intervening in the embedding of the model. We illustrate this by selectively limiting the ideological information in the embedding and evaluating recommendations. Second, our work points to a potential lack of definition in regulation and guidelines limiting the use of sensitive data (such as political orientation) in recommendations, whenever sensitive attributes can be learned independently by the machine without the need for engineers to specify them in the design or to provide them explicitly as input data.

## Data and methods

The following section details the empirical framework underlying our study. We begin by describing the panel of Twitter users for which ideological positions are available, along with the engagement signals and demographic attributes used as input data. We then present the matrix factorization recommender system we train on this data, and the latent dimensions it produces. Finally, we introduce the explainability metrics we develop to interpret these dimensions, and the attenuation procedure we use to intervene on the learned representations. Throughout this article, we refer to Supplementary Information (SI) for additional technical details on the data, methods, and results. The SI in S1 File is contain sections (identified by letters) that mirror the structure of the main text and figures. It is intended to provide the reader with the full methodological and empirical detail necessary to reproduce our findings, without disrupting the flow of the main narrative.

### Panel of Twitter users with ideological positions

To enable the analysis of ideological leaning in the spatial representation of a recommender system trained in our study, we start our study with a population of Twitter users for which we have ideological estimates. For this, we leverage a previously existing large dataset of French Twitter users collected during 2019 [32]. In this dataset, the positions of users are computed by applying a multidimensional ideology scaling procedure on follower networks, and calibrating positions with political survey data for statistical identification of the ideology parameters (using the Chapel Hill Expert Survey data [39]). Supplementary Information SI-A in S1 File present more details about this dataset, and SI-B in S1 File details the steps of the estimation of political positions. The dataset provides two political dimensions, deemed by authors of that study as the first two independent political dimensions that most determine the follower network: a Left-Right dimension, and a dimension measuring attitudes (negative and positive) towards elites and institutions (a dimensions commonly used in population studies). Both dimensions are calibrated so that political parties are positioned between values 0 and 10: 0 being far-Left and 10 far-Right, and 0 being no negative attitudes towards elites and institutions, and 10 being very negative attitudes (the distribution of the users over those two dimensions is shown in SI-Fig S1 in S1 File).

The users in our panel are highly self-selected in the sense that they chose to follow political figures on the platform. Prior work has established that such users exhibit high levels of political sophistication [40] and that their behavior can be adequately modeled through ideological positions. Moreover, our sample drawn randomly from a substantially large population of 361,831 users who followed a sufficient number of political figures. Our population is therefore representative of the ideologically engaged core of the political sphere on the platform.

### Engagement signals with news media content

We also leveraged a second dataset by the same authors [38], of 40.000 randomly selected users (among the initial 361.831 followers of MPs), and their last 3.200 tweets or posts. 50 users among these 40.000 had deactivated their accounts or the availability of their data via API. From this corpus of tweets, we identify those that contain a URL pointing to a web domain outside Twitter. This dataset contains 23.534.803 tweets pointing towards 426.014 domains. These web domains constitute the items to be recommended in our experimental framework. By training a recommender with these engagement trace data, we calculate which web domains could potentially be relevant to users. The details about the data collection and the design choices are presented in the supplementary information SI-A in S1 File.

### Demographic attributes of users

When analyzing how ideological positions might be encoded in the model of the recommender system, we consider several covariates, including demographic attributes. The dataset from which we obtained the ideological positions of users also includes the text of their profile bios on the platform. We further downloaded the profile photos of all users in the dataset. We use the M3 inference model [41] to infer the age and gender of these users by feeding it the text and photos from their profiles. From our dataset, we gathered information on gender and age for all the users having available bios on their profiles (89.5% of users). We also used M3 to determine whether accounts belonged to individuals or organizations. Among accounts of users in our panel, 17% were identified as organization profiles. Among the rest of the users, identified as individuals, the ratio between females and males is of 27.9%/72.1%, with the percentage of female individuals decreasing with age. Additional details of this procedure and quality metrics are included in SI-G.

### Matrix factorization recommendation algorithms

Next, we consider a recommender system aimed at predicting future user choices based on past engagement data. We consider that each user u∈𝒰 (X users, then Twitter) selects items i∈ℐ (domains of URL shared) a number of times denoted as *r*_*ui*_ (number of times that contents from a domain were shared). Our goal in training the recommender system is to predict which new item the user will select. Items the user has not yet interacted with (*r*_*ui*_ = 0). One of the most common algorithmic families for this task is collaborative filtering (CF), based on the principle that users that are similar in how they have engaged with content in the past will find the same similar recommended content relevant [42]. Collaborative filtering algorithms have also a key advantage that we leverage in our research design: they do not need any user or item attributes as input, and rely solely on engagement data. Because of this advantage, collaborative algorithms are also widely deployed in real-world settings [26].

We select the Non-Negative Matrix Factorization (NMF) [43] collaborative filtering algorithm for the recommendation problems. We call $R\in {\mathbb{R}}_{+}^{n\times m}$ matrix representing the interactions *r*_*ui*_ between users and items, and we infer a user matrix $P\in {\mathbb{R}}_{+}^{n\times k}$ and a item matrix $Q\in {\mathbb{R}}_{+}^{m\times k}$ such that their product approximates the observed data *R* of past interactions between users and items:


$$\begin{array}{c} R\approx P\cdot Q^{T} \end{array}$$ (1)

By computing a factorization as matrices *P* and *Q* we obtain a latent spatial representation, or embedding. In this representation or embedding of dimension *k* << *n*, the coordinates of users and items are encoded in these two matrices respectively. Because of the formulation linking the factor matrices with observations *R*, the model scores relevance as an inner product in the *k*-dimensional space of the embedding as ${\hat{r}}_{ui}={\text{p}}_{u}\cdot {\text{q}}_{i}$.

After having trained the model with our data, we evaluate its usefulness as its capacity to produce relevant recommendations, which we cast as the evaluation of the accuracy with which it predicts observations from our data. We evaluate the accuracy of our algorithm using the Hits@10 metric, which measures, for each user, the proportion of correct (i.e., previously observed in the test set) recommendations among the first 10 most relevant recommendations as per inner product value [44]. The training includes regularization parameters to avoid overfitting, the evaluations are made by cross-validation, and the hyper-parameters (*k*, initialization technique, used norm, solver...) are optimized by Particle Swarm Optimisation [45] to maximize the test accuracy. The best accuracies were obtained for *k* = 12 latent dimensions; this number largely depends on the size of the dataset, with lower or higher values of *k* leading to under or overfitting. Details on the pre-processing, the initialization [46], the solver used for factorization (multiplicative update [47]), and the mathematical formalization of the algorithm are available in Supplementary Information SI-C in S1 File. The random guess performance for our task is around 10^−4^. After training, our model reaches an accuracy of Hits@10 = 0.35, which is in line with the reported performance of real-world systems [48].

Our choice of computing synthetic recommendations comes with advantages and limitations. Computing recommendations ourselves with a model that we run locally, allows us to have full control of the parameters and observability of the representation spaces of the recommender. On the other hand, while our choice for recommender follows algorithmic principles adopted in the industry, our simplified model has considerable difference with real-world models operating on social media platforms. A recent study made the opposite design choice, choosing to inspect real recommendations shown to users on X to reconstruct the machine representation space in search for information pertaining to ideological positions of users [49]. This choice allows for more realism in the recommendation mechanism, but at the cost of having less observability of the model (authors can only approximately estimate the machine representation model), and for a comparatively limited number of users. In this regard, these two studies (the present one and that reported on [49]) are complementary and point to the same conclusions: the corresponding analyzed algorithms trained with X/Twitter data have information on ideology positions of users in their machine representation spaces.

### Latent dimensions of the recommender system model

The matrices *P* and *Q* define what is commonly referred to as a latent space, or embedding: a low-dimensional geometric space in which both users and items are assigned coordinates (their embeddings) that summarize their patterns of interaction. Two users with similar engagement histories will tend to occupy nearby positions in this space, as will two items that tend to attract the same users. The inner product between a user’s and an item’s coordinates then serves as a measure of their mutual affinity, i.e., the predicted relevance of that item for that user. The main advantage of training the recommender algorithm, instead of relying on recommendations served on the platform, is that we now have access to the model computed during training. The data used and the model trained have a much smaller scope than any real recommender system operating on the platform, but serve the purpose of showing that even extensively reduced scopes may still allow ubiquitous algorithms (such as matrix factorization) to learn sensible features. We call the positions of users and items in the latent space of the recommender, respectively, $P\in {\mathbb{R}}_{+}^{n\times k}$ and $Q\in {\mathbb{R}}_{+}^{m\times k}$. Each line *p*_*u*_ or *q*_*i*_, respectively *P* and *Q*, represents the coordinates of the user *u* (or the item *i*) in the embedding. For our setting *k* = 12, we denote $L_{0},L_{1},...,L_{11}$ the latent dimensions of the model of the recommender.

### Explaining the dimensions of the model of the recommender

To endow this latent or embedding space of the model of the recommender system with meaning, we develop an explanation model derived from attribution and hidden-layer semantic methods [33]. Attribution explanation methods aim to identify and quantify the contribution of each feature of entities (i.e., users and domains) to the prediction made by a model. In other words: How do changes in ideological positions of users affect their recommendations? Hidden layer semantic explanation methods aim to interpret the meaning of dimensions in the learned spatial representations. In other words: How does the ideology of users relate to position along the dimensions of the trained model? We consider the political positions as *implicit* input features, i.e., features of the users that are linked with the input features but that are not explicitly provided to the model during training.

With respect to the several available methods for attribution and embedding explanation and their ontologies (see the surveys by Tintarev et al. [36], Zhang et al. [33], and Marcinkevics et al. [28]), we develop a method that is: *global* (explaining the general structure of the model rather than singular recommendations [28]), *passive* (explaining the model after training [33]), *user-based* (leveraging user explanation rather than item explanation [36]), and *non-linear* (accounting for non-linear contributions of the features to the model). Section SI-D in S1 File of the Supplementary Material contains further details, positioning our explanation method within the literature.

#### Attribution explanation.

An attribution indicator quantifies the contribution of an input feature to the model’s prediction by comparing the result with and without this feature [50]. We propose an adaptation of this principle to continuous implicit input features [51] (see Supplementary Information SI-E in S1 File), which we will apply to the continuous latent dimensions of the recommender system model resulting from matrix factorization.

Let us denote as *L*_*j*_ a position in the latent space of the recommender along dimension j=0,…,11. We name Φ a feature of relevance for the explanation (e.g., position of individuals on the Left-Right or the anti-elite scale). The *local attribution factor* of a latent dimension *L*_*j*_ to an input feature Φ is defined as:


$$\begin{array}{c} S_{j}(\phi )=\frac{𝔼\left[L_{j}|(\Phi =\phi )\right]-𝔼[L_{j}]}{\sigma_{j}} \end{array}$$ (2)

The local attribution factor measures how the difference in input Φ results in a difference in the latent embedding *L*_*j*_ (i.e., how *L*_*j*_ reacts to Φ). It can be positive or negative depending on the direction of the effect. It is null if the latent dimension and the input feature are independent. Its scale is relative to the standard deviation $\sigma_{j}$ of the distribution of entities in the model along the considered latent dimension *L*_*j*_.

While the local attribution factor captures the sensitivity of a latent dimension to a specific value ϕ of a feature, it does not summarize the overall relevance of that feature across the full range of its possible values. To obtain a single scalar summarizing how much a feature Φ globally determines positions along dimension *L*_*j*_, regardless of where on the scale users fall, we aggregate the local attribution factor over the full range of Φ. We can average the local value to get the attribution of the whole feature defining the **global attribution factor** of *L*_*j*_ to Φ as the absolute average of the local attribution factor:


$$\begin{array}{c} S_{j,\Phi }^{\left(\text{Global}\right)}=\frac{1}{\phi_{max}-\phi_{min}}\cdot \int_{\Phi }|S_{j}(\phi )|\;d\phi , \end{array}$$

where $\phi_{max}$ and $\phi_{min}$ are the maximum and minimum values of the input feature. For example, an algorithm attributing impact to political positions is one where users with different political positions receive different recommendations.

**Fig 1:**
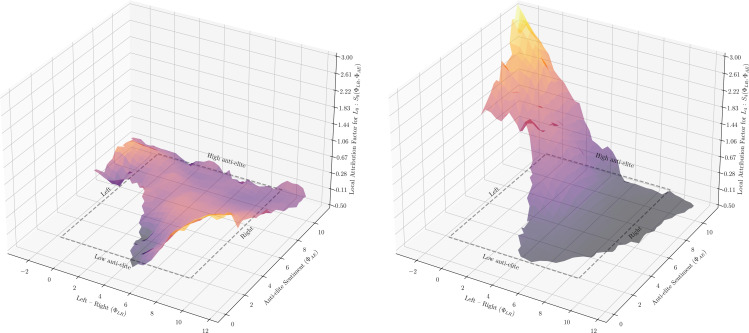
Example of the *local attribution factor*, *S*_0_ (left figure) and *S*_4_ (right figure) for two latent dimensions of the representation space of the recommender systems *L*_0_ and *L*_4_, on the feature space of users subtended by our two available political dimensions: Left-Right $\Phi_{LR}$ and Anti-elite sentiment $\Phi_{AE}$. The difference in local attribution factor illustrates different cases, where political features have no explanatory power (*L*_0_ on the left figure), and where some values of political features have explanatory power (*L*_4_ on the right figure).

Section SI-E in S1 File of the Supplementary Material provides additional details on the way this factor is estimated, the design choices made, and the comparison with alternative methodologies. Fig 1 shows these indicators comparing independent variables with dependent ones. We also employ the discrete version of the local and global attribution factor for categorical attributes of users and items (see SI-F in S1 File for details).

### Attenuating selected dimensions of the recommender model

One of the potential applications of the results of our study is the possibility of selecting particular dimensions of the recommender model (identified with a given attribute of users), and attenuating its importance or weight in the computation of recommendations. This application, which will be described and analyzed in the results section, is of interest in platform moderation, whenever platform policy has interest in removing or attenuating the influence of specific attributes.

Let $P\in {\mathbb{R}}_{+}^{n\times k}$ be our user embedding and $Q\in {\mathbb{R}}_{+}^{m\times k}$ our item embedding, and let *J* be a set of dimensions of this embedding that we wish to attenuate. We define the *reduced embedding* $\tilde{P}$ and $\tilde{Q}$ as, ∀u∈𝒰, ∀i∈ℐ and ∀j∈[0,k−1]:


$$\begin{array}{c} \tilde{P_{uj}}=\left\{ \begin{array}{cc} (1-\alpha )P_{uj} & \text{ if }j\in J,\\ P_{uj} & \text{ otherwise}, \end{array}\right.\\ \tilde{Q_{ij}}=\left\{ \begin{array}{cc} (1-\alpha )Q_{ij} & \text{ if }j\in J,\\ Q_{ij} & \text{ otherwise}, \end{array}\right. \end{array}$$

where α is a reduction factor ranging from 0 (base embedding) to 1 (fully reduced embedding). This reduced embedding defines a reduced model (with its scalar product).

#### Political leaning and diversity of recommendations.

To evaluate recommendations, specifically those resulting from reduced models in which selected dimensions have been attenuated, we evaluate accuracy of recommendations along side two key properties: political leaning (the mean ideological position of recommended domains or media outlets) and diversity (how heterogeneous the set of these recommended items is in terms of its ideological composition). We estimate the political position of each item *i* by computing the mean political positions of the users sharing the item weighted by the strength of the interaction *r*_*ui*_. We note $\mu_{\text{LR}}(ℐ)$ and $\sigma_{\text{LR}}(ℐ)$ the mean and the standard deviation of the Left-Right position of the items.

Let us call Rec[*u*] the set of items recommended to *u*. In our case study, we set recommendations to 10 items per user. We denote the Left-Right **leaning of recommendations** for a user *u* as


$$\begin{array}{c} ℬ(u)=\frac{\mu_{\text{LR}}(\text{Rec}[u])-\mu_{\text{LR}}(ℐ)}{\sigma_{\text{LR}}(ℐ)}. \end{array}$$

By construction, the leaning is negative for Left-leaning recommendations and positive for Right-leaning recommendations, with a scaling relative to the standard deviation of random recommendations.

We denote the Left-Right **diversity of recommendations** for a user *u* as


$$\begin{array}{c} 𝒟(u)=\frac{\sigma_{\text{LR}}(\text{Rec}[u])}{\sigma_{\text{LR}}(ℐ)} \end{array}$$

By construction, diversity spans from 0 to positive values, 1 meaning that recommendations are as diverse as the diversity of the available recommendable domains.

#### Web domains from media outlets.

To evaluate how recommendations constitute different media diets, we further identify which web domains in our dataset correspond to sites of media outlets and classify them into political categories. To do this, we leverage a pre-existing dataset of websites of the main 440 French media outlets, assorted into different categories, developed by Cointet et al., 2021 [52]. The authors of this dataset categorized media outlets by first identifying their main web domains, then crawling the HTML code of the main page and sub-pages, identifying hyperlinks connecting articles between different outlets, to finally compute a community clustering of the resulting citation network, identifying political categories with cluster. The full list of categories is in SI-Table S4 in S1 File. Among the media outlet categories produced by that study, four of them speak to Left-Right ideological groups of interest for our study: “Left Wing”, “Hyper center” (the most cited and traditional outlets), “Right Wing” and “Identitarian” media outlets. Further details on the definition and how we use these categories to evaluate the outcomes of recommendations for different attenuation of dimensions of the recommender system model are in SI-I.

### Ethics statement

Our study did not involve experimentation with human subjects. All the platform data used for the computation of attributes of users was collected using the API acces of X. The processing of political attributes data was declared on 19 March 2020 and 15 July 2021 at the registry of data processing at the *Fondation Nationale de Sciences Politiques* (Sciences Po) in accordance with General Data Protection Regulation 2016/679 (GDPR) and X policy. For further details and the respective legal notice, please visit the web page of project EPO: medialab.sciencespo.fr/en/activities/epo/. The processing of the data has been approved by the Research Ethics Committee of the Paris Institute of Political Studies in its decision nº2023−038.

## Results

In the last section we presented a recommendation algorithm trained from X sharing data, and a method allowing to explain the latent space of this algorithm. In this section we present the result of the explanation on our specific panel of users. We first quantify the explanatory power of political and demographic features over the latent dimensions of the trained recommender. We then validate this by mapping users with salient positions in the latent space back onto the political feature space, and corroborate our findings using an independent dataset of categorized media outlets. Finally, we evaluate the effect of selectively attenuating the ideologically-loaded dimensions on the leaning, diversity, and accuracy of recommendations.

### Explanatory power of political dimensions

**Fig 2:**
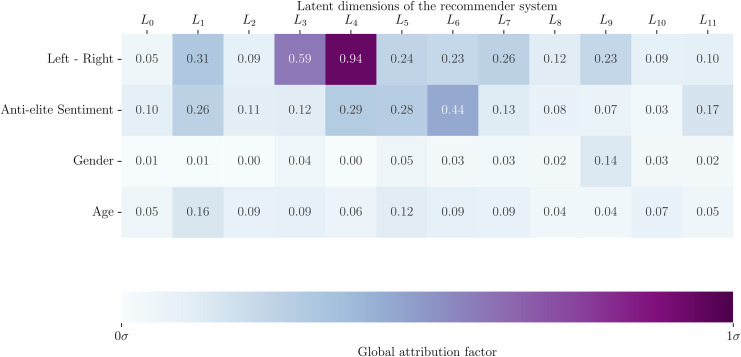
Explanatory power of user features (Left-Right position, Anti-elite sentiment, age, and gender) for each dimension of the latent space of the trained recommender system. Explanatory power is measured with the *global attribution factor*, measuring how much the dimensions would vary if the features were ignored. We identify *L*_3_ and *L*_4_ as the dimensions with the highest attribution for Left-Right ideology positions.

We first measure how our two political features (positions of users on the Left-Right scale and on the Anti-elite scale) relate to the recommendations that we compute. To do so, we compute the global attribution factor of Left-Right position $\Phi_{LR}$, Anti-elite sentiment $\Phi_{AE}$. We also compute how the other features, age and gender, might also participate in explaining recommendations by computing their global attribution factor. Fig 2 shows the global attribution factor of each dimension for each feature of individuals in the population of our study. The attribution factor (presented in data and methods) indicates, on average, how much a feature determine the position of users in the latent dimension of the recommender. We observe that a few latent dimensions relate to variations in Left-Right ideology and Anti-elite sentiments, while most dimensions do not relate to variations in gender and age. *L*_3_ and *L*_4_ stand as the recommender dimensions with the highest global attribution factor (0.59 and 0.94 respectively) for the Left-Right dimension, while *L*_6_ and *L*_4_ stand as the recommender dimensions with the highest global attribution factor (0.44 and 0.29 respectively) for the Anti-elite dimension.

These results are, however, largely consistent with other statistical metrics of dependency, such as Mutual Information, Pearson and Spearman correlation, Kendall Tau, and the chi-square test (see SI-Fig S2 in S1 File). Figure SI-Fig 47 in S1 File of the Supplementary Information highlights this difference by showing the specific regions of the political spectrum that are most related to the latent embedding. This locality allows for an additional distinction in how dimensions *L*_3_ and *L*_4_ capture political attributes: while *L*_3_ captures the degree to which individuals lay to the Left-wing side of the political spectrum, *L*_4_ captures how individuals lay to the Right-wing side of the spectrum. This delivers the first clue as to how these algorithms may model implicit features such as political leaning. Rather than capturing Left-Right positions as a single dimension in the recommender system model, the regularity of groups on the political Left and the political Right is captured independently as dimensions of the latent space.

Gender and age are comparatively unrelated to positions in the latent space, with the highest global attribution factors being 0.16 for age and 0.14 for gender.

### Observing latent attributes of the recommender in the feature space

**Fig 3:**
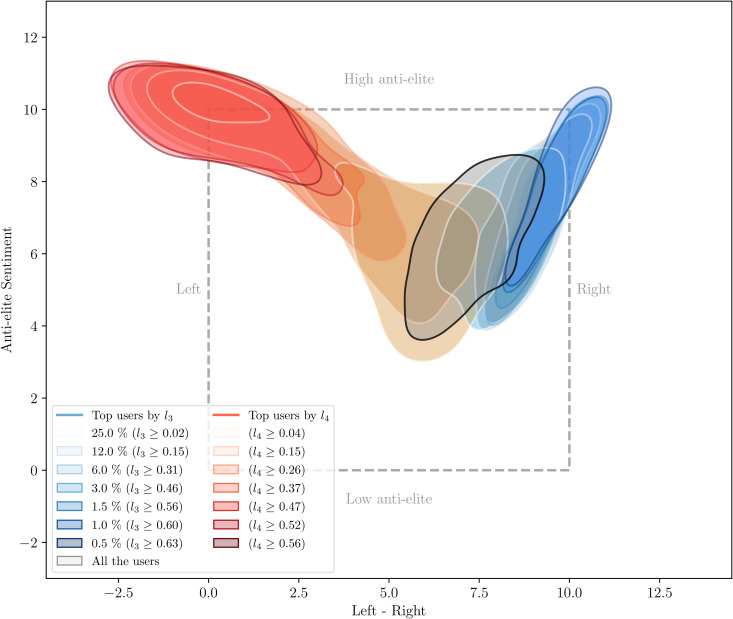
Distribution in the political feature space, for individuals with salient positions along dimensions *L*_3_ and *L*_4_ of the recommender latent model (adapted from [38]). *L*_3_ encodes, in the recommender system, the degree to which individuals lean to the political Right, while *L*_4_ encodes the degree to which individuals lean towards the political Left. Level curves correspond to likelihood values of 0.5 for the Kernel Density Estimation (KDE) of the subset of users with positions along latent dimensions *L*_3_ and *L*_4_ that are higher than the thresholds *l*_3_ and *l*_4_, respectively.

To validate this hypothesis, we proceed to map how the positions of individuals, according to their locations in the recommender’s latent space, are arranged within the feature space. To do so, we proceed in the following manner. For each one of the two latent dimensions that are most explained by Left-Right positions according to our attribution metric (*L*_3_ and *L*_4_), we select a set threshold values, allowing us to consider different quantile of individuals with the most salient positions along these dimensions (quantiles 0.5%, 1.0%, 1.5%, 3.0%, 6.0%, 12.0%, and 25.0%). Each quantile determines a threshold of cutoff value in each latent dimension. For instance, quantile 25% selects 25% of individuals with the most salient position (by definition), which for, e.g., *L*_3_ are those that have a value above *l*_3_ ≥ 0.02. Fig 3 (adapted from [38]) shows the distribution of the different groups of users in our study for different thresholds applied to positions *L*_3_ and *L*_4_. For each threshold, Fig 3 shows the level curve of the Kernel Density Estimation (KDE) of the resulting sample at likelihood value equal to 0.5. As hypothesized, these two dimensions capture the degree to which users are considered by the recommender system as being Left- or Right-leaning. We observe that this specialization also leans towards increasing Anti-elite positions. An examination of the dataset of bi-dimensional political positions that we use shows that this is because, for this population, but also more generally in French political, far-Left and far-Right positions are both strongly correlated with Anti-elite positions [32]. See Section SI-B of the SI for a characterization of the dataset showing this correlation. We present in SI-E.4 additional metrics used to measure political leaning and diversity in the latent space of the recommender.

In their scientific publication, X details how following, sharing, and engagement signals are used to train a multidimensional spatial representation of users and content [53]. Once this representation is computed, recommendations are generated by ranking the results of inner products between user-content pairs. This is the same mathematical principle underlying the matrix factorization approach employed in the present study, and constitutes the primary motivation for our methodological choice. Once a spatial representation has been learned, the multidimensional positions of users and items are organised into matrices, and matrix multiplications compute all pairwise inner products. The critical distinction between our approach and that of the platform lies in how these multidimensional representations are derived — both in terms of the input signals and the objective function optimised during training, the latter being necessarily dependent on the data signals available. X has access to the full breadth of behavioral signals generated on the platform. Our study, by contrast, is based on a specific, acquirable data signal that constitutes a subset of those available to the platform. If applying the same underlying mathematical principle — recommendation via pairwise inner product ranking — to a fraction of the platform’s available data is sufficient to produce a systematic correlation between ideological positions and positions in the recommender’s representation space, incorporating additional behavioral signals, as the platform’s own system does, would likely preserve this alignment with Left-Right ideological structure.

We conducted the same analysis for the latent dimension for which the second political dimension, the Anti-elite dimension, has the largest explanatory power: *L*_6_. Figures S9 and S10 in S1 File of the SI show comparatively how salient values along *L*_3_ and *L*_4_ identify individuals with salient Left- and Right-leaning positions, while salient values in the recommender model along dimension *L*_6_ identify individuals with very low negative attitudes against elites.

### Explanatory power of media outlets categories

Because both users and web domains (including websites of media outlets) are embedded in the latent space of the recommender, we can also assess whether dimensions *L*_3_ and *L*_4_ encode Left-Right leanings using new and independent data: the categories of media outlets. For this, we leverage the dataset developed by Cointet et al., 2021 [52] described in the Data and Methods sections. Because in this dataset the domains of media come endowed with categorical descriptors (crucially, relevant categories such as Right-Wing and Left-Wing media), we use the categorical version of our attribution factor, proposed in the Data and Methods section.

Using our categorical attribution factor, we measure which media categories best explain the position of web domains along dimensions *L*_3_ and *L*_4_. For *L*_3_, we find the first two categories with the highest value in attribution factor are “Identitarian” and “Right-Wing” media outlets (with attribution factor values 1.18σ and 0.81σ respectively, followed by category “Revolutionary Right” with value 0.32σ). For *L*_4_, one category has a much higher attribution factor than the rest: “Left-Wing” media outlets (with attribution factor value 1.77σ, followed from afar by “Local” outlets with value 0.48σ). Section SI-J of the SI provides further details on the measurement and visualization of these results.

These results show that dimensions *L*_3_ and *L*_4_ capture Left- and Right-leaning positions of users (shown in the previous section with positions from the dataset developed by Ramaciotti et al. 2022) and also coherently of media outlets (shown in the present section using categories of media outlets developed by Cointet et al., 2021). This convergent reliability of measurements provides strong evidence of the link between dimensions *L*_3_ and *L*_4_ of the representation space of the recommender and Left-Right ideological positions.

### Controlling for socio-demographic attributes

While dimensions *L*_3_ and *L*_4_ encode the ideological features of individuals and recommendable contents, an important question is whether they also encode additional relevant features that might be highly correlated with ideology. In the most extreme case, if ideology is completely correlated a socio-demographic attribute (e.g., age or gender), the recommender might learn dimensions for all these features jointly, in which case it would be fair to call into question the role of ideology in machine representation in our case study. To assess the independence of learned political representations, we control for attributes of users that might be correlated with ideology: age, gender, occupation, and language.

To identify occupations, we used the official standardized ontology developed by the French National Bureau of Statistics (*Institut National de la Statistique*, INSEE), called *nomenclature PCS* [54] (for *Profession et catégories socio-professionnelles*, or socio-professional categories in English). We collected the 3,000 most frequently-occurring words and bi-words in the Twitter bios of our user set and manually identified all words and bi-words referring to occupations. Inspired by the methodology of Sloan et al. [55], we then matched those occupations with the PCS categories and identified five main occupation classes with the highest number of matches in our dataset (accounting for 20.5% of all users in our study): 1) information, arts and entertainment, 2) business, IT and administration professionals, 3) elected officers and political representatives, 4) professors and higher scientific professions, and 5) legal professions. While the long-tailed distribution of users across PCS categories leaves open the possibility that unaccounted socio-professional categories retain some residual explanatory power in the representation learned by the recommender system, we note that any such category would by definition account for a very small fraction of the user population — one that falls well below the fractions for which the model already identifies distinctive left- and right-wing categorizations, as shown in Fig 3. Table S2 in S1 File in Section G of the SI provides a detailed characterization of these socio-professional categories.

We first observe that ideological positions and socio-demographic attributes are not independent among users in our population of studies. Users aged 29 and younger are significantly to the left of older users (aged 30 and older), with younger groups displaying systematically more anti-elite sentiments. Similarly, female users are to the left of male users, while both gender groups hold similar anti-elite sentiments. Section H.2 of the SI provides additional characterization of age and gender distributions in our panel in comparison to their ideological positions. Because of these dependencies of attributes in our population, we seek to establish the degree to which the main identified recommender system dimension linked with politics (*L*_3_ and *L*_4_) might also be capturing correlated features. We do this using the local attribution factor, testing, for each socio-demographic attribute, how sensible are positions of users along the dimensions of the recommender. To leverage positions of media outlets in our analysis of independence, we also compute the local attribution factor for the positions of media outlets using the media outlets categories from Cointet et al. 2021. We find that the age and gender attributes do not have a significant impact in determining positions of users along *L*_3_ and *L*_4_. The most determining attributes for the position of users and items in these dimensions are the media categories from Cointet et al. 2021. The most determining socio-demographic attributes for users’ positions along *L*_3_ and *L*_4_ are language (attribution factor = 0.45), age (attribution factor ≤ 0.3) and interest in ecology (attribution factor = 0.4; all attribution factors in absolute value), well below attributions factors measured for Left-Right positions (0.59 for *L*_3_ and 0.94 for *L*_4_; see Fig 2). The most salient attribution factors for socio-demographic attributes for all dimensions of the latent space of the recommender are available in SI Fig, S34 to S45 in S1 File.

In line with a prior literature, we treat the Left-Right dimension as the politically relevant ideological axis. This section examined whether other individual attributes may account for Left-Right positions — and for the positions that emerge in the recommender’s internal representation, or more precisely, in the behavioral data generated by users on which the recommender is trained. The set of potential confounding variables that might jointly explain both ideological positions and content-sharing behavior is, in principle, vast. The standard methodological response is to control for theoretically relevant covariates. We opt to test against variables known to correlate substantially with ideological positions, namely combinations of age, gender, and occupation [56–58]. While our approach yields insight into the role of ideology in shaping the behavioral traces that feed into algorithmic representations, it inevitably carries some limitations, as no finite set of controls can fully exhaust the confounder space. We nonetheless argue that understanding how geometric representations produced by algorithms relate to users’ ideological positions — and how those positions shape recommended content — constitutes a valuable contribution in its own right, independently of whether unaccounted confounders remain. Regardless of the causal pathways through which individually determined attributes (such as interest in sport or science) come to shape user behavior, the outcomes of greatest concern to researchers and regulators are those with political consequences. Our study cannot, as is true of any observational study, establish whether Left-Right positions causally determine positions in the geometric representation space of the recommender. What we do establish, however, is a quantifiable relationship — documented here for the first time — between users’ ideological positions and their positions within the recommender’s representation space, together with the downstream consequences this entails, while controlling for a range of variables that could plausibly serve as alternative explanations.

### Selectively attenuating the weight of features in recommendations

Our results so far allow for the identification of the role of ideology of users and contents in algorithmic recommendation, disentangling the role of ideological features from that of other socio-demographic variables. The importance of this result lays in the fact that political ideology or leaning is a key variable of interest in studies of online polarization, informational segregation, and algorithmic biases. This begs the question: If we can isolate the role of a relevant feature such as ideological position, can we also modify the recommender to avoid using it? In this section, we test this design principle by selectively attenuating the latent dimensions of the recommender that are associated with Left-Right ideological stances. We apply the procedure described in the Data and methods sections, selecting $J=\{L_{3},L_{4}\}$ as the dimensions to be attenuated (by a factor α∈[0,1]), yielding reduced embeddings $\tilde{P}$ and $\tilde{Q}$ for users and domains.

**Fig 4:**
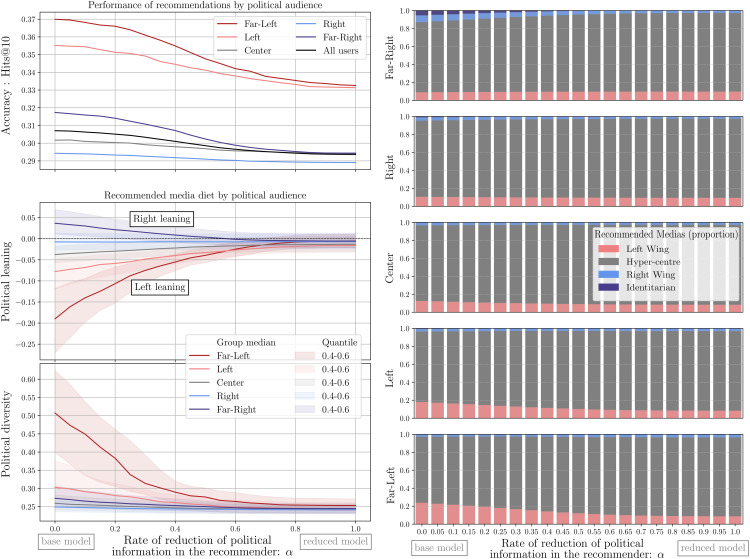
(Left-side panel) Effects on accuracy, leaning and diversity of recommendations, of selectively attenuating ideological dimensions in the latent space of the recommender by a factor α, and disaggregated for 5 groups of users (Far-Left, Left, Center, Right and Far-Right users). We observe a reduction in accuracy, accompanied by a decrease in left and right leaning, as well as a decrease in the left-right diversity of the recommendations. **(Right-panel)** Impact of attenuating ideological latent dimensions on the proportion of opinion media recommended for each political group. We see that tweaking reduces the left and right-wing media in favor of more mainstream media. Because mainstream media are still prevalent also for extreme users, then the reduction of political dimension ends up in a reduction of political diversity.

To test the effect of this selective attenuation experiment, we compute recommendations for values of α spanning from 0 to 1, by changing its value in steps by increments of 0.05 (i.e., 21 steps in total). For each value of α, we compute recommendations as the top-k ranked domains resulting from the inner product between $\tilde{P}$ and $\tilde{Q}$ (with k = 10), and we evaluate: the accuracy (Hits@10, or the proportion of correctly guessed items among 10 recommendations), the Left-Right leaning (the mean Left-Right position of the recommended domains), and the diversity (the standard deviation of the Left-Right positions of the recommended items) of recommendations. Both leaning and diversity are measured comparatively, relative to the leaning and the diversity of the overall item dataset, and are presented in the data and methods section. To increase the granularity of our analysis, we report on these metrics aggregating users by ideology groups, defined as follows: *Far-Left* (with Left-Right positions equal or below 2, i.e., $\phi_{LR}\leq 2$), *Left* ($2<\phi_{LR}\leq 4$), *Center* ($4<\phi_{LR}\leq 6$), *Right* ($6<\phi_{LR}\leq 8$), and *Far-Right* ($\phi_{LR}>8$). This binning relies on the fact that the Left-Right scale we use has been calibrated with political survey data in which position 5 stands for the political center, with 0 being the far-Left and 10 the far-Right positions. The values of accuracy, leaning, and diversity of recommendations for these five ideology groups, and for attenuation ranging from 0 to 1, are reported in Fig 4 (left-side panel).

Attenuating the weights of dimensions encoding Left-Right leanings in the latent space of the recommender system model amounts to destroying information learned during training, which results in our experiments in penalized accuracies, as measured by the rate of previously-chosen items that the recommender is able to guess. The effects of this decrease in accuracy are, however, marginal and heterogeneous: for different groups, loss of accuracy ranges from 0.02 (for the Right-leaning group) to 0.11 (for the Far-Left group). At the same time, in terms of the leaning of recommended contents, all groups shift gradually, from a situation in which they are recommended domains aligned with their ideological leaning, to one in which they get recommended centrist content. This shift is not, however, explained by the inclusion of more diverse content in recommendations, but rather by the increasingly centrist-focused content in recommendations, as evidenced by the decreasing diversity of recommended items as attenuation progresses with increasing values of α.

These results suggest that users initially consume (and thus get recommended) a mixture of content that is ideologically-aligned to their own positions, and other media, typically centrist main stream media. As we attenuate the importance of dimensions that encode ideologically-specific interests of users (*L*_3_ and *L*_4_), increasing α from 0 to 1, the importance given to these ideologically-aligned media decreases, leaving mostly centrist main stream in the set of recommendations. To test this hypothesis, we display in Fig 4 (right-side panel) the recommended media diet for each group, coloring recommended items with the media categories obtained from the dataset developed by Cointet et al., 2021, described in the Data and Methods sections. Fig 4 (right-side panel) shows that the share of ideologically-aligned recommendations decreases in favor of recommendations of media from the category called Hyper-center (the most central and mainstream media outlets in France). This phenomenon is also shown in more detail in SI-Fig S46 in S1 File displaying the distribution of the most recommended media for the Far-Left ideology group as α increases.

## Discussion and conclusions

Policy-makers, regulators, industry actors, as well as civil society actors grant increasing importance to algorithmic transparency, particularly in social media and in regards to phenomena related to politics (such as, e.g., polarization or informational segregation). Auditing recommendations is a relevant tool, but, in comparison with AI explanability, it often fails to speak directly to outcomes translatable in algorithm design principles. AI explainability, is, however, exceedingly challenging, because of the data required (as with audits), but also because of the complexity of the –black box– models used in recommendations. In this article, we proposed a way to reduce this complexity to take the first steps at causally analyzing how algorithms learn, leverage, but also *forget* (through our attenuation experiments) sensitive or relevant information.

In comparison to other studies, our strategy mixes a semi-synthetic approach (to both instantiate our experiments in realistic settings with real-world users, and to gain access to the model of a relevant recommender system by training our own), with computational experiments, leveraging two data resources: a dataset of Twitter users with estimated ideological positions, and a dataset of recommendable media outlets with categorical variables related to ideological groups. While we acknowledge the loss on realism in the recommendation mechanisms in our study, our choice of recommender algorithm represents both an ubiquitous mathematical principle and common practice in industry. In this regard our approach provides a novel approach in comparison with other studies by retaining complete control and observability of the trained model of the recommender.

Our results show that, in our setting (arguably simplified with respect to reality) common and ubiquitous recommendation algorithms trained on a subset of the data that real-world recommenders use, are able to learn political orientations of individuals that are not significantly correlated with socio-demographic covariates. While our setting is simplified, it is relevant because it shows that even simple components (i.e., ubiquitous matrix factorization algorithms) and partial data (shared content) might reproduce similar behavior, namely, autonomous machine learning and representation of ideologies. Both the platform’s recommender system and the one employed in our study rely on inner product ranking to select content for recommendation. The principal difference lies in the breadth of data signals available to the platform relative to those we use to train the recommender model. While our study draws on a specific data signal, we argue that the integration of additional signals would likely preserve the emergent Left-Right structure in the recommender, for two reasons. First, users’ Left-Right positions will also influence the other behavioral signals they produce on the platform. Second, spatial models of this kind are precisely designed to capture and disentangle multidimensional phenomena — making them well-suited to recover ideological structure even when it co-exists with other behavioral dimensions. In other words, if a fraction of the data available to real recommenders (user-domain choices), and if a simple commonplace principles (such as matrix factorization), can pick up ideology signals autonomously, it is probable that larger and more complex systems might do the same. In light of this insight, our work proposes the first step into measuring this phenomenon, and in a way that opens the path towards design principles tackling this.

Our results also open a novel avenue for social science research on human-AI interactions. The users in our panel are self-selected in the sense that they chose to follow political figures on the platform. Rather than constituting a limitation, this property makes them particularly well-suited for the present study: prior work has established that such populations exhibit high levels of political sophistication and that their behavior can be adequately modeled through ideological positions. Our sample was moreover drawn randomly from a substantially large population of 361,831 users who followed a sufficient number of political figures. Our population is therefore representative of the ideologically engaged core of the political sphere on the platform.

Our study focuses on a specific behavioral signal generated by these users, namely the sources they cite. Some degree of ideological homophily in cited sources is to be expected in our data. By training a recommender system on these data and recovering ideological structure in its learned representation space, we do not merely demonstrate coherence between ideological positions — estimated through the ideology scaling of following networks — and cited sources, which is not the primary focus of our study. Rather, we illuminate the degree to which, and the mechanism by which, ideological positions emerge within trained recommender systems. While several prior works have theorized the possibility of such a phenomenon, the present study provides a controlled, step-by-step experimental setting in which this theoretical process can be empirically observed.

It is also worth noting that the phenomenon we reproduce concerns ordinary users, and is thus distinct from other forms of algorithmic bias documented in the literature, such as models learning the ideological valence of words, documents, or political figures. Our work therefore contributes to both social science and computer science research on the complex feedback dynamics characterizing human-AI interaction. If these interactions can be broadly described as a chain of influence — from internal opinions to behavioral trace data, to algorithmic learning, to recommendations that in turn shape opinions — our work provides a new experimental framework for studying how individual opinions come to influence algorithmic systems.

Finally, our experiments with attenuated political information stored in the trained recommender system also show that it is in principle possible to selectively remove information available to the recommender learned during training (even when it was autonomously learned), and that this affects recommendations marginally in terms of accuracy while drastically changing the leaning and diversity of recommendations. While we do not call for platforms (or any other actor for that matter) to norm and decide which content from which ideology should reach which groups among the public, computing recommendations inevitably equates to imposing such norms, knowingly (if engineers integrated it in the design) or unknowingly (if the recommender learned ideologies independently). Our results are a step forward in making clear what norm is being implicitly put forward by algorithms and how.

## Supporting information

S1 File Supplementary information document. (PDF)
