# Supplementary material for: How do recommender systems learn political opinions? A semi-synthetic step-by-step experiment
Source: PLoS One. 2026 May 26;21(5):e0349341. doi: 10.1371/journal.pone.0349341 (PMC13210316; doi:10.1371/journal.pone.0349341)
Supplement: S1 File — (PDF) [file pone.0349341.s001.pdf]

Supplementary Information

How do recommender systems learn political opinions? A semi-synthetic step-by-step experiment

Tim Faverjon[a,b], Jean-Philippe Cointet[a], and Pedro Ramaciotti[c,a,b,*]

[a]médialab Sciences Po, Paris, France
[b]Learning Planet Institute, CY Cergy Paris University, Paris, France
[c]Complex Systems Institute of Paris Ile-de-France ISC-PIF CNRS, Paris, France
[*]Corresponding author: pedro.ramaciotti-morales@cnrs.fr

ABSTRACT

Recommendations play a crucial role in shaping informational diets on social media, raising concerns regarding potential consequences such as political segregation. We take an algorithm explanability approach, as opposed to a description of recommendations, to show how recommenders inadvertently create geometrical representations of the ideological position of users with minimal and ubiquitous platform data, and how this impacts content diets. In comparison to work showing the existence of ideological structures in machine representations, we provide a step-by-step causal explanation of their formation. To achieve this, we compute synthetic recommendations with a model trained on real-world data from a panel of nearly 40 thousand X users and the contents they shared on the platform. We show that elementary recommendation principles trained on content dissemination data produce a spatial representation of the Left-Right positions of users in our panel in the recommender, which is also independent of other common attributes, such as age and gender. We explore the consequences of our findings by modifying these ideological representations in the recommender and analyzing the trade-off in resulting recommendations in terms of political leaning, diversity, and relevance of offered contents.

Contents

A User-item dataset . . . 2
User collection • Item dataset collection
B Political attitude estimation . . . 2
C Recommendation algorithm . . . 3
Mathematical formalization • Pre-processing • Metrics • Solver • Performances
D Political explanation method : design choices . . . 6
Advantages of Latent Embedding Explanation • Advantages of Global Explanation • Advantages of User-based Political Explanation • Advantages of Post Training Explanation • Comparison with existing embedding explanation methods
E Political explanation method . . . 7
Local attribution factor • Estimation of the attribution factor • Global attribution factor • Validation of the explanation. • Visualisation of latent leaning and diversity
F Categorical features explanation method . . . 10
Discrete Attribution Factor
G User demographic features . . . 12
Language • Occupations • Status qualifiers • Topics of interest • Keyword Age • Keyword Gender • Machine Learning Inference : Age, Gender and Organization
H Statistical dependence among demographic features . . . 16
Co-occurence of occupations, qualifiers and topics • Relationships between political positions and Demographic Features
I URLs belonging to known medias . . . 18
J Socio-demographic explanation . . . 18
Figures **19**
References **40**

Methods

A User-item dataset

We chose to collect users following French members of parliament on Twitter because the literature allows us to estimate their political attitude[1]. We consider this dataset a case study for recommending content to real-world users using elementary recommendation principles.

A.1 User collection

The user collection method :

- We consider the French Members of Parliament (MPs) in May 2019: 813 out of 925 were officially on Twitter and had allowed the collection of their followers.
- We collect the 4.487.430 unique followers of the MPs.
- We remove the user following less than 3 MPs[1] and the user with less than 25 followers[2].
- We ignore all the users following the same subset of MPs (e.g., if three users are following exactly the same MPs, we keep only one of them)[3].

We obtain that way 368.831 users.

A.2 Item dataset collection

To simulate our recommendation algorithm, we need data to consider as an item to recommend. Since views and likes cannot be widely scrapped from X, we consider the URLs shared by Twitter users as items. We then consider sharing an URL on Twitter as a meaningful user-item interaction.

- We randomly extract 40.000 users from the 368.831 followers of MPs (presented in section A.1).[4]
- We collect, among their last 3.200 tweets or posts, all the tweets containing URLs.
- We process the URLs (using ural library - tool from[3]) in order to recognize distinct internet domains (e.g., lemonde.fr, lefigaro.fr, businessinsider.de...).

We obtain, after this collection, 39.950 users, 426.014 domains, and 23.534.803 sharings. We will consider the URLs as *items*, and we will call *interaction* between a user u and an item i the number of times that i has shared u (if u has never shared i we say that there is no interaction).

B Political attitude estimation

Estimating individuals' political opinions has been a long-standing challenge for political scientists. One limitation is the difficulty in defining a political opinion (e.g., is that the individual's vote ? Or what he will answer if asked ? Or a predisposition of agreement ?). Theoretical works from psychology introduced the notion of *attitude* as a potential solution to this problem[4,5]. We call *political attitude* a state of mind or a predisposition, positive or negative, toward a determinate politically related statement. This notion allows researchers to consider political ideology as a latent random variable that influences behavior, allowing them to estimate this value formally.

Some works have already leveraged political attitude estimation on social media with USA-centered data, using the political representative followed by each user as a meaningful trace of political attitude[2,6]. However, this kind of model was difficult to export in the European case of the figure because of the complexity of the European politics[57].

Nonetheless, recent work by Ramaciotti Morales[8] has expanded this model, showing that it is possible to infer the political opinions of certain users also in the case of more complex political spaces. Using political expert data such as the Chapel Hill Expert Survey (CHES)[9], which ranks each European party according to their political attitudes[6] towards a set of 51 different issues (e.g., European integration, special rights for minorities...), it is possible to estimate the political attitude of these users.

[1]The political attitudes estimation would be too uncertain for those users

[2]This *good habit* is proposed by[2] to remove part of the fake accounts

[3]This choice is a simplification used by Ramaciotti; hence, those users are considered as equal for the attitude prediction method. We can consider the user chosen as an average representative of the group of users.

[4]We choose to consider 40.000 users to make scale economies of resource (to collect data, train the algorithm, and analyze the result) while keeping a reasonable number of users in order to obtain statistically significant results. 50 users among these 40.000 had deactivated their accounts or the availability of their data via API.

[5]USA political debate tends to be mono-dimensional while for most European countries this debate has 2, 3 or 4 main dimensions

[6]i.e., their positive or negative predisposition towards certain political statements or positions

We hypothesize that following an MP carries a certain ideological weight, which is greater the more distant the ideological positions of the user and the MP are. In particular, we define the probability that user i follows an MP j as :

$$P(A_{ij} = 1|\alpha_i, \beta_j, \gamma, \phi_i, \phi_j) = logit^{-1}(\alpha_i + \beta_j - \gamma|\phi_i - \phi_j|^2)$$

Were $A_{ij} = 1$ when user i follows MP j, α_i and β_j are the “activity” (tendency to follow others) and “popularity” (tendency to be followed) of users i and j, ϕ_i and ϕ_j are their latent ideological parameters, and γ is a normalization constant.

These latent ideological parameters can be approximated for a large set of users and MPs by a Correspondence Analysis (CA) of the adjacency matrix $A \in \mathbb{R}_+^{|Users| \times |MPs|}$[10]. Thus allowing the MPs and their followers to be projected in a multidimensional ideological space. The ideological dimensions thus obtained correlate with the political analysis carried out by the experts (CHES), but they lack a consistent measurement scale, which prevents, on the one hand, identifying the political "center" and "extreme" and, on the other hand, comparing the results obtained dimension by dimension or country by country. To overcome these limitations, we apply an affine transformation to the ideological space in order to transpose it into an attitudinal space whose dimensions correspond to the attitudinal dimension of the CHES. The CHES classifies each European party according to its attitudes towards a set of 51 different political issues. Thus, the affine transformation is optimized to approximate the parties’ positions in the ideological space as accurately as possible.

We can see fig. S1 the resulting spatial distribution of the user dataset on the two main attitudinal dimensions of the French political spectrum: Left-Right and Anti-elite sentiment (CHES dimensions). We observe that the average political attitude in our dataset is $\mu_{LR}(\mathscr{U}) = 6.4$ and $\mu_{AE}(\mathscr{U}) = 6.7$, and that some users go beyond the reference frame of CHES. This happens because the frame is made at the EU level, specifically for party attitudes and not user attitudes. Our sample is more to the Right, Anti-elite, than the average EU party position.

Several empirical studies have shown the consistency of this estimation technique[11,12]. In particular, we can show that the users’ auto-declarations in their bios are coherent with their position in the attitudinal space.

C Recommendation algorithm

C.1 Mathematical formalization

The Non-Negative[7] Matrix Factorization (NMF) problem is a known problem in mathematics[13]. In the recommender literature, we can see many different formulations of the problem. For our work, we used the formulations proposed by Lee et al.[14] and adapted to the recommendation case by Mnih et al. and Koren et al.[15,16].

Let $\mathscr{U}$ be a set of n users and $\mathscr{I}$ be a set of m items.

For each user and item $u, i \in \mathscr{U} \times \mathscr{I}$, we call r_{ui} the rating of the user u for the item i.

The problem of recommendation consists of predicting future ratings from a known set of ratings.

We call $\mathscr{K} \subset (\mathscr{U} \times \mathscr{I})$ the set of known ratings (one of the particularities being that while we have several ratings for each user, the number of known ratings is really small compared to the unknown ratings: $|\mathscr{U}| < |\mathscr{K}| << |\mathscr{U} \times \mathscr{I}|$)

Let $R \in \mathbb{R}_+^{n \times m}$ be the rating matrix. The rating matrix can be defined as follows[8]:

$$\forall (u,i) \in (\mathscr{U} \times \mathscr{I}), R_{ui} = \begin{cases} r_{ui} & \text{if } (u,i) \in \mathscr{K} \\ 0 & \text{otherwise} \end{cases}$$

Let $k \in \mathbb{N}^*$ be a small integer such that $k << \min(n,m)$. The NMF problem is to find $P \in \mathbb{R}_+^{n \times k}$ (Users matrix) and $Q \in \mathbb{R}_+^{m \times k}$ (Items matrix) positives matrices, such that $R \approx P \cdot Q^T$.

Because the equality is proved impossible for $k < \text{rank}(R)$, we then refer to the optimization problem :

$$\min_{P \in \mathbb{R}_+^{n \times k}, Q \in \mathbb{R}_+^{m \times k}} \left\| R - P \cdot Q^T \right\|_2^2$$

[7] The nonnegative constraint is not systematic in all matrix factorization recommender systems, it is usually justified in problems where we seek to extract only positive influence of latent variable on interactions, and it is often said to avoid over-fitting problems

[8] Note: we consider unobserved interactions as null interactions.

Adding regularization terms to avoid overfitting, we get :

$$\begin{aligned}\min_{P\in\mathbb{R}_+^{n\times k},Q\in\mathbb{R}_+^{m\times k}} & 0.5\cdot\left\|R-P\cdot Q^T\right\|_2^2 \\ & +\alpha_P\cdot l_1\cdot k\cdot\|P\|_1 \\ & +\alpha_Q\cdot l_1\cdot k\cdot\|Q\|_1 \\ & +0.5\cdot\alpha_P\cdot(1-l_1)\cdot k\cdot\|P\|_2^2 \\ & +0.5\cdot\alpha_Q\cdot(1-l_1)\cdot k\cdot\|Q\|_2^2\end{aligned} \tag{1}$$

Where α_P, α_Q, l_1 are parameters respectively controlling regularization on P, on Q and the norm used. $\|\cdot\|_1$ and $\|\cdot\|_2$ refer to the usual norms 1 and 2 on matrix spaces.

In this formalization, k is the number of dimensions in the algorithm's latent embedding, P is the coordinate of the users in the embedding, and Q is the coordinate of the items in the embedding. Once the embeddings P and Q have been computed, we can predict the new ratings by a simple dot product: $\hat{R}=P\cdot Q^T$.

C.2 Pre-processing

To improve the performance of the algorithm, we apply some pre-processing to our dataset (collected section A.1).

First, we eliminate from our data set all the generic URLs referring to social media (e.g. X.com, facebook.com...)[9]. In fact, those URLs give us little information about the shared content (because we consider only the domain of the shared URLs) while being among the most shared ones. They then risk having a significant impact on the loss function with low improvement in accuracy. Furthermore, because most users share those URLs, their interactions carry little information. Leaving them out of the algorithm changes little to the algorithmic explanation results, besides faster computation time and more accurate predictions. We, in fact, observed higher accuracy scores without those URLs.

Second, we recursively remove all the users who have shared fewer than 10 different URLs and the URLs shared by fewer than 10 different users. We chose this threshold (of 10) for three reasons :

1. We build our test set from 20% of the total interactions, having at least 10 different interactions for each user or item in the full data set ensure us a reasonable probability to have at least one interaction for each user or item in the test set.[10]
2. We choose to evaluate our model with a Hit@10 metric (presented section C.5); for this reason, we try to reduce the number of users with less than 10 interactions in the train set.
3. We want to avoid the cold start problem, well-known for collaborative filtering methods.

Note that because the removed users and items were marginal, their removal had a negligible impact on the loss function (and the algorithmic result) while having a considerable impact on accuracy and computation time.

Our interactions matrix is implicit, i.e., it does not indicate a direct rating from a user to an item but only the number of times that user shared this item. This is a drawback for our recommender, because we want to predict the ratings of the users in order to recommend the favorite content. This is a common problem while developing matrix factorisation recommender systems[17], because implicit interactions usually do not scale linearly with the appreciation[11].

Calling $r_{ui}^{(\text{imp})}$ the implicit rating (i.e. the number of time a user u shared the item i), we estimate the rating r_{ui} by applying a log scale :

$$r_{ui}=1+0.98\cdot\log(1+2\cdot r_{ui}^{(\text{imp})})$$

The log scale is usually recommended to go from implicit to explicit ratings[18], and the parameters (0.98 and 2) have been chosen depending on the data set in order to get ratings in a 1 to 10 range.

This step avoids the risk of having few interactions with an enormous impact on the loss function, while most of the other interactions are unimportant. We can notice that because the log is monotonous, the ordinal accuracy metrics (such as $Hits@10$ that we use) stay unchanged.

After the cleaning phase we obtain a rating matrix $R\in\mathbb{R}_+^{29.373\times 32.639}$ with $n=29.373$ users, $m=32.639$ items, and $|\mathscr{K}|=3.277.738$ ratings[12].

[9]Full list: X.com (5.158.473 sharings), facebook.com (1.127.131), youtube.com (889.057), instagram.com (260.816), linkedin.com (91.444), amazon.com, bit.ly, fllwrs.com, unfollow.fr

[10]During the evaluation, if no item is in the test set, the accuracy is considered 0. We can then expect a real accuracy higher than the measured one.

[11]clicking five times on a content does not mean it is evaluated 5 times better than another

[12]This represent 0.34% of the possible interactions

C.3 Metrics

To test our performance, we build from our interaction data set $\mathscr{K}$ a train set $\mathscr{K}^{\text{train}}$ (with 80% of the interactions) and a test set $\mathscr{K}^{\text{test}} = \mathscr{K} \setminus \mathscr{K}^{\text{train}}$ (20% of the interactions).

Because in real-world applications, the recommendation systems are used mainly to order or classify content, we choose to use an ordinal accuracy metric. Specifically, the $Hits@K$ metric, widely used in the literature[19]. This metric aims to consider the recommendation problem as a binary classification problem, in which we seek to predict the K best contents for a given user and return the F1 score of this classification.

Let's call $\mathscr{T} := (\mathscr{U} \times \mathscr{I}) \setminus \mathscr{K}^{\text{train}}$, the set of all the possible new interactions (i.e., the interactions not in the train set).

For each user $u \in \mathscr{U}$, the possible items of u among $\mathscr{T}$ can be define as :

$$\mathscr{T}^i(u) = \{i \in \mathscr{I} \text{ s.t. } (u,i) \in \mathscr{T}\}$$

Let $u \in \mathscr{U}$, and $K \in \mathbb{N}_+^*$. The set of the K best **predicted** items for u among $\mathscr{T}$ is :

$$Rec@K(u,\mathscr{T}) := \{i \in \mathscr{T}^i(u) \text{ s.t. } |\{j \in \mathscr{T}^i(u) \text{ s.t. } \hat{r}_{uj} \geq \hat{r}_{ui}\}| < K\}$$

The set of the K best **real** items for u among $\mathscr{T}$ is :

$$Pref@K(u,\mathscr{T}) := \{i \in \mathscr{T}^i(u) \text{ s.t. } |\{j \in \mathscr{T}^i(u) \text{ s.t. } r_{uj} \geq r_{ui}\}| < K\}$$

We then define the metric $Hits@K$ as the proportion of top K items guessed for u :

$$Hits@K(u,\mathscr{T}) = \frac{|Rec@K(u,\mathscr{T}) \cap Pref@K(u,\mathscr{T})|}{K}$$

$$Hits@K(\mathscr{T}) = \sum_{u \in \mathscr{U}} \frac{Hits@K(u,\mathscr{T})}{|\mathscr{U}|}$$

This metric is, in fact, the $F1-score$ of the classification problem of finding the best K new items for each user. In this work, we will use $K = 10$ mainly because of the size of our data set. Note that if a user has only $K_0 < K$ non-zero interactions in the test set, we then consider the $Hits@K_0$ metric instead (this concerns a minority of users).

Note that because $\mathscr{T} = (\mathscr{U} \times \mathscr{I}) \setminus \mathscr{K}^{\text{train}}$ we are not only trying to predict the best contents among the test set, but among all the possible interactions. The random guess performance of this task is $Hits@10(\mathscr{T})[\text{Random Guess}] \approx 10^{-4}$.

C.4 Solver

We solve the optimization problem with the *Multiplicative Update solver*. Proposed by[14] this solver is a gradient descent with adaptive learning rate solver, which allow fast convergence and positivity of the solution. Concretely at each step the solver update Q and P as follow :

$$P_{ui}^{t+1} \leftarrow P_{ui} \cdot \frac{(RQ)_{ui}}{(PQ^TQ)_{ui}}$$

$$Q_{ui}^{t+1} \leftarrow Q_{ui} \cdot \frac{(P^TR)_{ui}}{(P^TPQ^T)_{ui}}$$

This algorithm is proven to converge toward a minimum and preserve the positivity of the solution.

We initialize the matrix P and Q with a *Non-negative Double Singular Value Decomposition* method[13] as proposed by[20], to ensure faster convergence, and better results[14].

To compute the training, we used the Python code proposed by[21].

We then optimize the hyperparameters with a *Particle Swarm Optimization* (PSO) algorithm. Introduced by[22], the PSO algorithm is a non-linear, non-convex optimization method commonly used for hyperparameter optimization. Among the hyperparameters, we find the normalization factors, the number k of latent dimensions, and an indication of the maximum number of steps of a multiplicative update to perform or the maximum loss to reach. We train the PSO algorithm on the hyperparameters, trying to optimize the cross-validation accuracy of the model on the $Hits@10(\mathscr{T})$ metric.

C.5 Performances

Our trained NMF algorithm with optimized hyperparameter by PSO performs with an accuracy of $Hits@10(\mathscr{T})[\text{NMF}] = 0.35$. This result compares positively to the estimated random guess and is in line with the reported performance of real-world systems[23]. From the algorithm, we get an embedding with $k = 12$ latent dimensions.

[13] The method is inspired from SVD but ensures positivity of the factorization.

[14] By accessing better local minima.

D Political explanation method : design choices

In this section, we present arguments justifying our algorithmic explanation methods. We highlight the advantages of using explanations rather than simple audits of results. We explain our design choices: a *passive*, *global*, *user-based* explanation methods that allow for the *attribution of implicit input features*[15]. We highlight the advantages of understanding the semantics of the latent embedding produced by the recommendation system. We compare our method with existing literature, keeping in mind that our method is meant to be only one of the multiple tools necessary to respond to the transparency demand on recommendation algorithms.

D.1 Advantages of Latent Embedding Explanation

Here are the advantages of explaining the latent layers of recommendation models compared to the audit of results:

- Generalization: Today, there is a wide diversity of recommendation systems on the market. While most systems integrate and present results differently (top 10 items, news feed, compatibility score, etc.), a large majority of models currently use one or more latent embedding layers, which consist of a multidimensional representation of users and items[24]. Therefore, a method capable of explaining the structure of those embeddings is likely more easily reusable than audit techniques dependent on the structure of results, even opening the door to potential comparisons between seemingly entirely different algorithms.
- Statistical significance: Algorithmic recommendations are usually predictions of user-item interactions; they are then situated in a very high-dimensional space ($d \geq \min(n,m)$ with n users and m items), whereas latent spaces have a much lower number of dimensions. Working on a lower-dimensional latent space not only facilitates obtaining statistically significant results but also avoids ignoring the majority of less-consumed content, which is difficult to count due to its low appearance in recommendations but nevertheless constitutes a significant portion of consumed content.
- Safety engineering: Being able to identify users' characteristics (such as political positions) in latent spaces also opens up the possibility of imagining methods to modify these latent spaces that act directly at the model level.

The feasibility of analyzing user embeddings as representative layers of algorithmic operation is supported by their typical proximity to the final prediction layers; embeddings are often separated from the output by straightforward, interpretable components (such as a dot product, in our case). However, this approach is less insightful for systems where embeddings are not situated near the final layers of the model.

D.2 Advantages of Global Explanation

Global explanation methods in algorithmic explanation aim to provide insights into a model's overall behavior by analyzing its structure, parameters, or predictions across the entire dataset. These methods (e.g., SHAP, LIME, surrogate models) summarize how input features influence predictions on average rather than focusing on individual instances.

Most recommendation algorithms face the so-called "cold start" problem, which states that predictions for some users will be challenging to make for new or marginal users due to the lack of data. One reason is that data from online social networks often shows a power-law distribution in user activity, as is the case in our dataset (i.e., a minority of users and items participate in most interactions). This effect has the consequence that most users (especially new users) have very few known interactions.

The cold start problem for new or inactive users is commonly addressed by using inferences that consider all users globally rather than each user separately (e.g., collaborative filtering methods). Using a global explanation method for recommendation algorithms helps better account for these global mechanisms while providing satisfactory explanations for users with low activity and newcomers.

In addition, we emphasize that our goal is to develop a method that identifies potentially large-scale systemic mechanisms, such as average political leaning, polarization, or content diversity, rather than individual biases. Although it is relevant to offer local explanations to improve the user experience, in our goal of minimizing risks for democracy and freedom in the digital public sphere, it is more pertinent to identify global phenomena.

D.3 Advantages of User-based Political Explanation

We argue that using a user-based explanation is more reliable than an item-based one. Several algorithm audit methods focus on identifying political content recommended by the algorithm[2]. However, sociology widely recognizes that cultural content consumption (even non-political) is related to political orientation. Therefore, the algorithm may recommend non-political content for political reasons. In order to take into consideration politicized[16] but non-political content, it is important to have a method focused on users rather than content.

[15] precisely, users' political positions

[16] Content whose appreciation is correlated with political positions and not with other controlled factors (such as socio-demographic factors) can be considered as politicized.

By relying on well-defined and measurable political positions of users, we avoid the difficult and blurry task of defining what content can and cannot be considered political.

D.4 Advantages of Post Training Explanation

We propose a post-hoc (passive) explanation method rather than an interpretable model for algorithmic governance purposes. Indeed, it is much more complicated for regulators to compel a platform to employ a particular interpretable model, whereas it is easy to impose explanation standards for systems already deployed by platforms.

D.5 Comparison with existing embedding explanation methods

The goal of our explanation method is to identify the impact of users' political positions on their position in the latent embedding of recommendation algorithms. We can, therefore, classify our method into the family of *attribution methods*. More specifically, in our case, we try to attribute the impact of *external* (or implicit) input features, as political positions and socio-demographic characteristics are not part of the explicit input data of the model but are implicitly linked with this data (i.e., sharing behavior depend in part on political and socio-demographic features).

There are already many attribution methods for explaining learning model embeddings.

- **Gradient methods**[25]: These methods use the gradient of the embedding with respect to variations in input features to attribute importance to each input feature. These methods have two limitations in our case: (1) they can only attribute the impact of explicit input features to the model, and (2) they depend on the ability to compute the gradient in question, which is not the case for collaborative filtering algorithms.
- **Decomposition and perturbation methods**[25]: The decomposition methods (typical of deep learning models) use a set of defined rules to decompose the signal arriving at the output of the model among the preceding layer, up until input features. The perturbation methods study the effect on the output of small perturbations in the input. These two methods can bypass the gradient problem (i.e., don't require computation of the gradient), but they still have the same limitation of being unable to attribute an impact to external features (i.e., feature not in the input).
- **Surrogate model methods**: These methods, which consist of constructing an interpretable surrogate model that links the embedding to a set of external characteristics, have the advantage of being applicable to external (or implicit) input data. However, to provide good results, it is necessary to have external characteristics that are rich and explanatory of the embedding in order to obtain satisfactory accuracy on the surrogate model[26]. As we do not have such data and, more generally, we want a method that is applicable without requiring a large amount of secondary data, we choose not to use these methods.

Because of the limits of existing explanation methods, we have chosen to develop a new method specific to recommendation algorithm political explanation tasks.

E Political explanation method

Previous work has already shown that it is possible to identify statistical relationships between recommendation systems' embedding dimensions and users' political positions[27]. Our objective is then not only to identify but also to characterize the specific political positions learned by the model and quantify the effect on the recommendations given.

We formalize this problem as an attribution problem: we quantify how differences in the embedding position can be attributed to differences in the political positions of the users.

E.1 Local attribution factor

Let's consider our recommendation algorithm Γ trained on Twitter data. Consider its latent embedding (of dimension $k = 12$) : $P \in \mathbb{R}_+^{n \times k}$ for users, and $Q \in \mathbb{R}_+^{m \times k}$ for objects.

Let Φ_{LR} and Φ_{AE} be random variables representing, respectively, the users' Left-Right attitude and Anti-Elite sentiment. Let $L_0, ..., L_{11}$ be random variables representing the latent dimensions of the embedding learned by the algorithm. Each user is considered a drawing of those random variables. We note l_j^u the jth latent position of user u, and ϕ_{LR}^u, ϕ_{AE}^u respectively the Left - Right and Anti-elite attitudes of user u. Φ_{LR} and Φ_{AE} take value in $[-5; 15]$ and $L_0, ..., L_{11}$ take value in $\mathbb{R}_+$.

We are trying to see if some characteristics of the users (in particular ϕ_{LR} and ϕ_{AE}) are influencing some latent dimensions $l_0, ..., l_{11}$. We can achieve this by studying the conditional distribution of the latent dimension depending on the political positions $f_{\text{Latent}|\text{Attitude}}$. There are several ways to measure the attribution of an input i to a result $m(\mathbf{x}(i))$. One common and general way is, given a model m that operates on a feature vector $\mathbf{x}$, to measure the influence of a feature i by calculating the expected difference in outcomes when feature i is randomly perturbed[26] :

$$\iota_{m,\mathbf{x}}(i) = \mathbb{E}_{y_i}[m(\mathbf{x}) - m(\mathbf{x}_{-iy_i})]$$

Where y_i is a random perturbation of the input feature i and $m(\mathbf{x}_{-iy_i})$ is the result of the model with the feature i perturbed. Nonetheless, this definition relies on the ability to measure $m(\mathbf{x}_{-iy_i})$ and is then restricted to explicit input features[17]. In our case, political positions are implicit input features: they are potentially related to the input data (URLs shared on Twitter) but are not part of it. We adapt this formula by considering the perturbation as resulting from a variation in political positions.

Keeping the idea of measuring the expected difference in outcomes between a when a model is perturbated, we introduce the *local attribution factor* of latent dimension L_j to political positions Φ_{LR}, Φ_{AE} as the expected difference in outcomes between a random user and a user having specific political attitude :

$$S_j(\phi_{LR}, \phi_{AE}) = \frac{\mathbb{E}[L_j|(\Phi_{LR} = \phi_{LR}, \Phi_{AE} = \phi_{AE})] - \mathbb{E}[L_j]}{\sigma_{L_j}} \tag{2}$$

Where σ_{L_j}, also written σ_j, is the standard deviation of L_j and is a scaling factor.[18]

The local attribution factor measures how the difference in input (Φ) results in a difference in the latent embedding (L_j). It can be positive or negative depending on the direction of the effect. It is null if the latent dimension and the input feature are independent.

Figure S5 we can see the local attribution factor of L_0 and L_4 depending on ϕ_{LR} and ϕ_{AE}. On the first hand, we see that $S_0(\phi_{LR}, \phi_{AE}) \approx 0$, indicating that L_0 is independent from Φ_{LR}, Φ_{AE} and hence not sensible to political positions. On the other hand we see that expected L_4 is higher for left wing users ($S_4(\phi_{LR}, \phi_{AE}) > 0$ for $\phi_{LR} < 4$) and reciprocally expected L_4 is lower for right wing user ($S_4(\phi_{LR}, \phi_{AE}) < 0$ for $\phi_{LR} > 6$). In this case, we can say that L_4 is reacting to the perturbation of Left-Right attitude, and we can precisely identify the regions of the political spectrum that have a higher impact on the dimension.

E.2 Estimation of the attribution factor

We estimate the expected L_j conditionally to the political positions by computing the local average of L_j. We define the *attitudinal neighborhood* of (ϕ_{LR}, ϕ_{AE}) as the set :

$$\begin{aligned}\mathscr{U}_{\phi_{LR},\phi_{AE}} = \{u \in \mathscr{U} \text{ s.t. } & \phi^u_{LR} \in [\phi_{LR} - \Delta\phi_{LR}, \phi_{LR} + \Delta\phi_{LR}] \\ & \text{and } \phi^u_{AE} \in [\phi_{AE} - \Delta\phi_{AE}, \phi_{AE} + \Delta\phi_{AE}]\}\end{aligned}$$

In our case we use $\Delta\phi_{LR} \approx 0.9$ and $\Delta\phi_{AE} \approx 0.7$.[19]

We compute $\mathbb{E}[L_j|(\Phi_{LR}, \Phi_{AE})]$ by measuring the average l_j of the attitudinal neighborhood :

$$\mathbb{E}[L_j|(\Phi_{LR} = \phi_{LR}, \Phi_{AE} = \phi_{AE})] \approx \overline{l_{j|(\phi_{LR},\phi_{AE})}} = \frac{\sum\limits_{u \in \mathscr{U}_{\phi_{LR},\phi_{AE}}} (l^u_j)}{|\mathscr{U}_{\phi_{LR},\phi_{AE}}|}$$

We compute $\mathbb{E}[L_j]$ as the average l_j of the whole user dataset :

$$\mathbb{E}[L_j] \approx \overline{l_j} = \frac{\sum\limits_{u \in \mathscr{U}} (l^u_j)}{|\mathscr{U}|}$$

Finally we compute σ_j with the classical estimator for the standard deviation :

$$\sigma_j = \sqrt{\frac{1}{|\mathscr{U}|} \sum_{u \in \mathscr{U}} \left(l^u_j - \bar{l}_j\right)^2}$$

E.3 Global attribution factor

While the local attribution factor is a meaningful indicator to attribute impact from some precise political positions, it could be helpful to measure the global attribution factor for summary purposes. We define the global attribution factor of a latent dimension L_j to an input feature Φ as the expected attribution factor given a random input feature value ϕ.

[17] i.e., features that are part of the model input

[18] We can note here that because we do implicit feature attribution and not explicit feature attribution, we do not expect the sum of the contributions to be equal to 1 such as for[28]. Therefore, our metric has no maximum and is scaled primarily for the sake of comparing contributions across different latent dimensions.

[19] In particular we choose $\Delta\phi_{LR} = \frac{1}{16}(max_{u \in \mathscr{U}}(\phi^u_{LR}) - min_{u \in \mathscr{U}}(\phi^u_{LR}))$ respectively ϕ_{AE} for $\Delta\phi_{AE}$

We compute the global attribution factor as the average of the absolute[20] attribution factor across the feature space.

$$S_{j,\Phi}^{(\text{Global})} = \frac{1}{\phi_{max} - \phi_{min}} \cdot \int_{\Phi} |S_j(\phi)| \, d\phi \tag{3}$$

Where ϕ_{max} and ϕ_{min} are the maximum and minimum values of the input feature. Non-sensible dimensions will have $S^{(global)} = 1$. An algorithm that attributes impact to political positions is one where users with different political positions receive different recommendations.

E.4 Validation of the explanation.

Once we identify which political positions influence which latent dimension, we may want to see how this influence impacts the embedding spaces.

We study the conditional joint distribution of the political positions Φ_{LR} and Φ_{AE} depending on the latent dimensions (formally: $f_{\Phi_{LR},\Phi_{AE}|L_i}(\phi_{LR}, \phi_{AE})$). Conditional groups of users with similar latent dimensions will receive similar recommendations. For each latent feature L_i we decide for a threshold l_i^* and consider the group of conditional users with $l_i^u > l_i^*$, on this group we measure 2 phenomena :

1. **The latent leaning**: which will measure how different the political positions of conditional users compared to those of random users, defined as $B_i(l_i^*) = \mathscr{B}(f_{\Phi_{LR},\Phi_{AE}|L_i > l_i^*}(\phi_{LR}, \phi_{AE}), f_{\Phi_{LR},\Phi_{AE}}(\phi_{LR}, \phi_{AE}))$ where $\mathscr{B}(\cdot,\cdot)$ is a statistical measure of the difference between distributions.
2. **The latent diversity**: which will measure how conditional users have diverse political positions compared to the political diversity of random users, measured $V_i(l_i^*) = \frac{\text{Var}(\Phi_{LR},\Phi_{AE}|L_i > l_i^*)}{\text{Var}(\Phi_{LR},\Phi_{AE})}$.

In the case where the latent features are independent from political positions, then we would have $f_{\Phi_{LR},\Phi_{AE}|L_i > l_i^*}(\phi_{LR}, \phi_{AE}) = f(\phi_{LR}, \phi_{AE})$ for all l_i^*, thus a leaning of 0 and a diversity of 1. If the distributions are different, the leaning will indicate how much the top users in the latent dimension are not representative of all users, and the diversity will indicate how similar the political positions of those users are.

A detail worth noting is that we study the conditional distribution knowing $l_j > l_j^*$, rather than $l_j^{(1)} < l_j < l_j^{(2)}$. This offers larger samples and, therefore, more statistically significant data, and works for non-negative latent dimensions where only positive effects are learned[13].

We refer to these measures as leaning and diversity because we argue that these measures are related to the actual leanings and diversity observed in recommendations.

Latent leaning. The leaning measures how different the distribution of political positions conditioned on a specific latent feature is from the base political distribution. There are many ways to quantify the difference between distributions. In this work, we will consider the means comparison because it is more interpretable and simpler.

The **means latent leaning** of a latent dimension L_j toward an input feature Φ is defined as :

$$B_{j,\Phi}(l_j^*) = \frac{\mathbb{E}_{\phi}\left[\Phi|(L_j > l_j^*)\right] - \mathbb{E}_{\phi}\left[\Phi\right]}{\sigma_{\Phi}} \tag{4}$$

It measures the political mean shift of top users in one latent dimension. It is null in the case of independence. It is relative to the standard deviation of the input feature, and it can be positive or negative depending on the direction of the leaning.

Latent diversity. The diversity measures if the users near to each other in a specific latent dimension are also similar to each other politically. This measure is relative to the base variance for scale purposes, allowing model comparison. We use variance here because, in our case study, the political positions are distributed similarly to normal distributions, which fits particularly well with variance measures. However, other diversity metrics could be suitable depending on the feature to be explained. Here, we use a threshold to define the conditional group of users because we want to study the diversity of positive latent dimensions. However, k-nearest neighbor groups (in the latent space) can also be used to assess diversity measures.

In one dimension we can define the political *latent diversity* of L_j, for a threshold l_j^* and a political attitude Φ as :

$$V_{j,\Phi}(l_j^*) = \frac{\text{Var}(\Phi|(L_j > l_j^*))}{\text{Var}(\Phi)} \tag{5}$$

[20] This computes both the negative and positive contributions across the input feature.

E.5 Visualisation of latent leaning and diversity

One can visualize latent leaning and diversity by plotting the different distributions of $f_{\Phi_{LR},\Phi_{AE}|L_i>l_i^*}(\phi_{LR},\phi_{AE})$ for various thresholds l_i^*.

Let l_j^* be a fixed thresholds for L_j and $p^* \in [0,1]$ a probability threshold. We define the *high-density area* $\mathscr{D}^{(j)}_{l_j^*,p^*}$ as the political positions area where the density of users with $l_j > l_j^*$ is higher than p^*. Formally :

$$\mathscr{D}^{(j)}_{l_j^*,p^*} = \{(\phi_{LR},\phi_{AE}) \text{ s.t. } f_{\Phi_{LR},\Phi_{AE}|L_j>l_j^*}(\phi_{LR},\phi_{AE}) \geq p^*\}$$

In figure S8 we show $\mathscr{D}^{(0)}_{l_0^*,p^*}$ and $\mathscr{D}^{(4)}_{l_4^*,p^*}$ for different thresholds l_0^* and l_4^* with $p^* = 0.5$.[21] We observe that users with high l_0 are similar to random users in terms of political positions (this is because L_0 is independent of political positions). And, as expected, we observe that users with high l_4 have a high probability of holding Left and Anti-elite attitudes (we say that L_4 lean toward Left and Anti-elite attitudes).

In figure S13, we now observe the latent bias. As expected from figure S8, $B_{0,\Phi_{LR}} \approx 0$ and $B_{0,\Phi_{AE}} \approx 0$, while $B_{4,\Phi_{LR}}$ grow toward Left and Anti-elite as the latent threshold grow.

The latent diversity of L_0 figure S14 shows us new phenomena invisible from the other metrics; while L_0 has no political leaning, we see for growing l_0^* values a decrease in diversity, showing that the dimension is specializing in ultra-center users. This phenomenon could not be detected by the attribution factor alone because of the high density of users in this region of the political spectrum, thus justifying the importance of observing diversity.

The latent diversity of L_4 figure S15, on the other hand, shows that for low l_4^* the dimension has a high diversity from all left and anti-elite. Still, this dimension goes on specializing in extreme left and anti-elite for high l_4^*. It is interesting to notice that with political positions (normally distributed) and latent features (distributed with high density near to zero and long tails), we expect latent features we strong political leaning to have high diversity for low l_i^* thresholds (as the population decreases abruptly and the mean move to the low-density political area during the transition phase) and have low diversity for high l_i^* thresholds (as the dimension specializes to a specific part of the spectrum), we can observe this transition phenomenon for L_4 figure S8.

For simplicity purposes, one can decide beforehand on a threshold to evaluate the leaning and the diversity of each latent dimension. We often choose to use a percentile threshold because we are not guaranteed that the scale of the different latent dimensions will be comparable.

We see the highest leanings for Left-Right figure S9 and for Anti-elite figure S10.

F Categorical features explanation method

We saw section E how to build an explanation from continuous implicit input features such as political positions. However, we see section H that political positions as well as users' sharing behaviors are statistically related to socio-demographic features. Therefore, to disentangle variance uniquely attributable to political positions from that captured by their socio-demographic proxies, we explicitly include socio-demographic features as controlling variables in our explanatory models. Two complementary strategies can be employed to isolate the variance uniquely driven by political positions from that captured by their socio-demographic proxies:

1. *Stratified attribution.* For each level (or category) of a given socio-demographic feature, estimate the political-attitude attribution factor separately. By comparing these stratum-specific estimates, you effectively "hold X constant" and see how much of the latent dimension is driven by politics alone.

2. *Proxy attribution.* Apply the same attribution algorithm to the socio-demographic feature itself. If this proxy yields a low attribution factor, it confirms that the variance attributed to political positions is independent of demographic confounds.

In this section, we adapt the attribution factor proposed section E for categorical features. This allows us to apply the proxy attribution strategy for socio-demographic features (such as occupation, age, or language...) in section J.

F.1 Discrete Attribution Factor

Let's consider a categorical input feature represented by the discrete random variable A (i.e. for all $u \in \mathscr{U}$, $A^u \in \Omega_A$). Let L_j be a latent feature of our recommendation system Γ with k latent dimensions ($j \in [|0,k-1|]$).

[21] $p^* = 0.5$ has been chosen for visualization reasons, we can see the same effect for other values of p^*

The *local discrete* attribution factor is defined by generalizing the local attribution factor for continuous variables (equation E.1) to the categorical case as :

$$S_j(a) = \frac{\mathbb{E}\left[L_j|(A=a)\right] - \mathbb{E}[L_j]}{\sigma_j} \tag{6}$$

The *global discrete* attribution factor is defined by generalizing the global attribution factor for continuous variables (equation E.3) to the categorical case as :

$$S_{j,A}^{(\text{Global})} = \frac{1}{|\Omega_A|} \cdot \sum_{a \in \Omega_A} |S_j(a)| \tag{7}$$

If A is a binary feature represented by a Bernoulli random variable, the attribution factors can be simplified as follows.[22]

Theorem 0.1 (Local Binary Attribution Duality)**.** *Let A be a Bernoulli random variable over user set $\mathscr{U}$, with $P(A=1) = \frac{|\mathscr{A}|}{|\mathscr{U}|}$ and $P(A=0) = \frac{|\mathscr{U}|-|\mathscr{A}|}{|\mathscr{U}|}$, where*

$$\mathscr{A} = \{u \in \mathscr{U} \mid A^{(u)} = 1\}.$$

Let L_j be the j-th latent dimension of a recommendation model, with standard deviation $\sigma_j > 0$. Define the local binary attribution factor for category $a \in \{0,1\}$ as:

$$S_j(a) = \frac{\mathbb{E}[L_j \mid A=a] - \mathbb{E}[L_j]}{\sigma_j}.$$

And the global binary attribution factor as:

$$S_{j,A}^{(Binary)} = \frac{1}{2} \cdot \sum_{a \in \{0,1\}} |S_j(a)|$$

Then the two factors satisfy the relation:

$$S_j(0) = -\frac{P(A=1)}{P(A=0)} S_j(1). \tag{8}$$

And the global binary attribution factor can be simplified as :

$$S_{j,A}^{(Binary)} = \frac{\left|S_j(1)\right|}{2\left(1-P(A=1)\right)} \tag{9}$$

Proof. We note $\mathscr{U}$ the set of all users and $\mathscr{A} = \{u \in \mathscr{U} \mid a^{(u)} = 1\}$ the set of users belonging to the category $A=1$.
We can then write :

$$\mathbb{E}[L_j] = \frac{1}{|\mathscr{U}|} \sum_{u \in \mathscr{U}} l_j^u$$
$$\mathbb{E}[L_j|A=1] = \frac{1}{|\mathscr{A}|} \sum_{u \in \mathscr{A}} l_j^u$$
$$\mathbb{E}[L_j|A=0] = \frac{1}{|\mathscr{U} \setminus \mathscr{A}|} \sum_{u \in \mathscr{U} \setminus \mathscr{A}} l_j^u$$

Furthermore, because A is Bernoulli and L_j is positive we have :

$$|\mathscr{U} \setminus \mathscr{A}| = |\mathscr{U}| - |\mathscr{A}|$$
$$\sum_{u \in \mathscr{U} \setminus \mathscr{A}} l_j^u = \sum_{u \in \mathscr{U}} l_j^u - \sum_{u \in \mathscr{A}} l_j^u$$

[22]The multi-category or overlapping multi-category case can be deduced as one vs all or one vs one.

We can then write the local binary attribution factor as :

$$\begin{aligned}
S_j(0) =& \frac{\mathbb{E}\left[L_j|(A=0)\right]-\mathbb{E}[L_j]}{\sigma_j} \\
=& \frac{1}{\sigma_j}\cdot\left(\frac{1}{|\mathscr{U}\setminus\mathscr{A}|}\sum_{u\in\mathscr{U}\setminus\mathscr{A}} l_j^u - \frac{1}{|\mathscr{U}|}\sum_{u\in\mathscr{U}} l_j^u\right) \\
=& \frac{1}{\sigma_j}\cdot\left(\frac{1}{|\mathscr{U}|-|\mathscr{A}|}\left(\sum_{u\in\mathscr{U}} l_j^u - \sum_{u\in\mathscr{A}} l_j^u\right) - \frac{1}{|\mathscr{U}|}\sum_{u\in\mathscr{U}} l_j^u\right) \\
=& \frac{1}{\sigma_j}\cdot\frac{|\mathscr{A}|}{|\mathscr{U}|-|\mathscr{A}|}\cdot\left(-\frac{1}{|\mathscr{A}|}\sum_{u\in\mathscr{U}\setminus\mathscr{A}} l_j^u - \left(\frac{1}{|\mathscr{A}|}+\frac{|\mathscr{U}|-|\mathscr{A}|}{|\mathscr{U}|\cdot|\mathscr{A}|}\right)\sum_{u\in\mathscr{U}} l_j^u\right) \\
=& \frac{|\mathscr{A}|}{|\mathscr{U}|}\cdot\frac{|\mathscr{U}|}{|\mathscr{U}|-|\mathscr{A}|}\cdot\frac{-1}{\sigma_j}\left(\frac{1}{|\mathscr{A}|}\sum_{u\in\mathscr{A}} l_j^u - \frac{1}{|\mathscr{U}|}\sum_{u\in\mathscr{U}} l_j^u\right) \\
=& -S_j(1)\cdot\frac{P(A=1)}{P(A=0)}
\end{aligned}$$

Both attribution factors (for $A=1$ and $A=0$) will have opposite signs; we can consider, by convention, that $S_j(1)>0$ and $S_j(0)<0$, renaming categories if needed.

Using this result, we can write the global binary attribution factor as :

$$\begin{aligned}
S_{j,A}^{(\text{Binary})} =& \frac{1}{|\Omega_A|}\cdot\sum_{a\in\Omega_A}|S_j(a)| \\
=& \frac{1}{2}\cdot\big(S_j(1)-S_j(0)\big) \\
=& \frac{1}{2}\cdot\left(S_j(1)+\frac{P(A=1)}{1-P(A=1)}S_j(1)\right) \\
=& \frac{S_j(1)}{2\big(1-P(A=1)\big)}
\end{aligned}$$

The *global binary* attribution factor is then :

$$S_{j,A}^{(\text{Binary})} = \frac{\big|S_j(1)\big|}{2\big(1-P(A=1)\big)} = \frac{\Big|\mathbb{E}\big[L_j|(A=1)\big]-\mathbb{E}\big[L_j|(A=0)\big]\Big|}{2\sigma_j} \tag{10}$$

□

Global binary attribution factor is well-suited to attribute even for highly unbalanced categories (which often occur for user features such as age group, occupation, interest, language, etc.). Global binary attribution factor is well-suited to attribute even for highly unbalanced categories (e.g., age group, language). In fact, for the local binary attribution factor, as $P(A=1)\to 1$ one obtains $S_j(1)\to 0$, effectively masking the over-represented class's contribution. By contrast, the global binary factor scales this dominant term by $2\big(1-P(A=1)\big)$, which also vanishes, thereby balancing impacts from both majority and minority classes without unduly emphasizing the smaller category. However, the binary attribution framework requires clearly defined positive and negative classes. In our keyword-driven approach, we can only detect the positive class (e.g., users self-identifying to an occupation) and cannot unambiguously define the complementary negative class (i.e., users who did not include any occupation keywords).

G User demographic features

We estimate user socio-demographic features to identify potential covariates linked with political positions. In this section, we present and discuss the methodology of estimation.

We collect the data available from our users' Twitter profiles: screen name, verified status, followers and friends counts, and description. We estimate the socio-demographical features of the users from the auto-declarative information available in their descriptions. In our dataset, 22.087 out of 29.373 users (75%) had an available description to collect. We can notice that

this value is higher than for the average Twitter users (around 48% of all users[29]) because the users in our dataset are only active users (i.e., sharing links and following MPs).

A Python package extracting all the information presented in this section from Twitter bios has been built and is available at twitter_profile_predictor.

G.1 Language

The first analysis we made was to estimate the language of each user. Giving the difficulty of the task on very small texts, we use three different available Python language detectors: `langid.classify`, `langdetect.detect`, and `cld3.get_language`, and we then get the correct language by majority vote or return *Unrecognized* if no language matches with another.

We can see table S1, the main languages used by the users. The great majority (92,4%) of the descriptions are French or English. Some users are non-french speakers; those users are here because they participate actively in the French political debate, following French MPs and being connected to the rest of the digital public sphere. We also observed that some items (URLs) are in non-French languages, and their sharing is related to user languages.

G.2 Occupations

Estimating Twitter users' occupation from profile information is a common task in computational social sciences, in particular in attempts to assess the socioeconomic status (SES)[30]. Because SES is usually considered a set of material, cultural, and social capital, it is then related to occupation. This makes the task of occupation prediction from Twitter descriptions reasonably common in the literature. The easiest way to estimate users' occupations is to use the auto-declared occupation in their description. According to[31], 20% of active users declare their occupational status on their description.

A common technique is to automatically search for occupations in the description, and link them to the standardized classifications like the *nomenclature des Professions et Catégories Socioprofessionnelles* (CSP2020) for France, the *Standard Occupation Classification* (SOC2020)[32] for UK or USA or the *International Standard Classification of Occupations* (ISCO-08)[33,34].

There are 4 primary difficulties of this task in the goal of assessing SES[34]. (1) Distinguish main occupations among hobbies or secondary occupations[23] (2) Access representativity on Twitter among the different classes: educated white men are over-represented among Twitter users. (3) Ensure that using a given occupation keyword is auto-declarative. (4) Wrong and/or biased Auto-declared occupations: High-status users and men are more likely to post their occupations on their bios, and the keywords used are often more laudatory than for national statistic surveys.

We tackle these problems by replacing the automatic detection of occupations with annotated occupations, focusing mainly on the most commonly declared occupations.

Here are our method's steps :

1. Tokenize the description, removing punctuation and stop words (in English and French).
2. Extract the most common words and bi-words.
3. Recognize the words (or bi-words) referring to auto-declaration of occupation (e.g., journalist, professor, city councilor...).
4. Link the declared occupation with the standard classifications (CSP2020, SOC2020, ISCO-08).
5. Check (by human verification) if using the keyword in bios actually refers to auto-declaration of occupations. And remove the keywords with accuracy under 80%.
6. Highlight the classes and sub-classes of occupations relevant to our analysis.

This technique not only allows us to obtain higher accuracy because of human verification, but mainly allows us to identify more users by adapting the method to our specific dataset.

Here's how we tackle the challenges cited before :

(1) In this work, we don't try to predict SES but only estimate types of occupations in order to link them with online sharing behaviors. Then, we do not care so much about the main occupation that determines the salary, and we can make the hypothesis that interest in some occupation will lead to similar content consumption, whether it's a primary or secondary occupation. We then chose to label multiple occupations for users who declared more than one.

(2) In this work, representativity is also less of a problem because we do not aim to make real-world scale statistics but to understand how the occupation of users influences the recommendation algorithm, specifically on X. The algorithm

[23] e.g., "Engineer and gardener on the weekend." Is that an engineer or a gardener? "Actor, producer, film director, and writer." What is the main occupation ?

recommends an unrepresentative environment, and we want to understand the impact of socio-demographic variation in this precise environment. We then stick to the unrepresentative population of Twitter.

(3) Statistical study presents 30% of misclassification by automatic keyword detection. The leading cause of misclassification is the use of qualifying words (e.g., president, manager, director... can both be occupations and qualifier-related keywords) and the use of occupational keywords in a nondeclarative framework (e.g., I hate journalists)[34]. For this, we rely on human verification and annotation, which brings us firstly to consider qualifiers words apart from occupation (see section G.3), and secondly to check manually the use of the keywords and keep only the one with accuracy over 80%[24].

(4) To avoid misclassification due to misleading declarations, we concentrate on the most used words and bi-words. This allows us to be highly specialized in our dataset and to adapt to specific ways of describing our population. And we choose to consider not specific occupations but large groups of occupations proposed by the official classification CSP. This ensures not only the statistical significance of the result but also reduces the risk of misclassification because of misleading declarations.[25] In any case, we note that we can only measure "declared" occupations, and good lies remain simply unrecognized.

Because our dataset is 80% French, we choose to analyze the occupations collected using the French standard classification CSP2020. Table S2 we can see the most important categories identified in our dataset. We can notice an over-representation of highly educated positions[26] from information, politics, and business. This bias has already been observed, and it's mainly due to the fact that workers from those categories are more likely to state their professional titles on their descriptions (among other things, because they use their profiles for professional purposes). This adds up to our sample bias already discussed.

We obtain this way at least one occupation for 20.5% of the users (this value matches the expectations of[31]).

G.3 Status qualifiers

In addition to occupations, Twitter users often use words to qualify their professional, personal or social status, we called here these word *qualifiers*. We used the same method as for occupations, examining the most frequent words and bi-words and identifying keywords qualifying a status associated with the user. We identified several types of qualifiers, some referring to professional identity, some to civil identity, some to political identity, ... Each identified qualifier corresponds to several specific keywords, verified by humans. We found at least one qualifier for 31% of the users. Here are the different qualifiers we have identified.

Professional qualifiers:

These keywords provide information about users' occupations, but this information indicates a position or qualifies a profession rather than indicating a sector or occupation. Here are the different identified qualifiers: advisor, director, president, founder, enthusiast, leader, assistant, manager, student, executive, delegate, alumni, candidate, vice president, professional, specialist, independent, elected, PhD, expert, important member, secretary-general, ambassador, collaborator, administrator, coordinator, doctor, board member, deputy director, apprentice, master, technician, worker. In particular, identifying these qualifiers allows us to avoid several misclassification problems related to occupations. We expect that some of these qualifiers will be correlated with users' political positions.

Type of Actor:

On Twitter, many accounts do not belong to individual users but to organizations. To distinguish one from the other, we identified a series of keywords characterizing the type of actor owning the account. List: association, official account, personal account, university, media, committee, federation, agency, startup, company, union, foundation, city hall, think tank. In particular, thanks to this qualifier, we can distinguish between official and personal accounts. We observed that these types of keywords are less precise than others for determining account ownership, but we judged them reasonable to use.

Affiliation to a Group:

These qualifiers indicate a declaration of affiliation to a political or cultural group. Here, the qualifiers do not specify which affiliation it is, only if it exists. List: Activist, member, citizen, supporter, fan, volunteer.

Degree and University:

In France, certain universities and degrees can impact identity, indicating membership in a social group. Among the most used words, we recognized some of these universities. List : Sciences Po, Sorbonne, Mines.

G.4 Topics of interest

As we are interested in the recommendation mechanism, it is sometimes valuable to know the interests of users in our dataset (e.g., politics, digital, communication, etc.), as these interests could strongly influence the consumed content. Using the same technique as for occupations, we have identified and classified keywords referring to specific topics in users' bios. These words are not intended to indicate the user's discipline, occupation, or profile but simply an affinity with a topic.

[24] For instance we notice that plural occupation keywords (e.g., "parliamentarians") most often don't refer to auto-declaration of occupation.

[25] e.g., While "digital project manager" and "senior software engineer" are two different occupations, they both are Business, IT, and administration jobs

[26] Cadres et professions intellectuelles supérieures

Here are the identified topics: politics, digital, communication, culture, public sector, innovation, law, education, tech, sciences, health, business, research, marketing, development, music, management, economy, environment/biodiversity, ecology, security, entrepreneurship, data, history, finance, press, energy, justice, agriculture, recruitment, social media, strategy, climate, real estate, construction, tourism, sustainable development, literature, mobility, diplomacy, agri-food, design, philosophy, sociology, engineering.

G.5 Keyword Age

Estimating users' age solely by keyword is particularly delicate because it is rarely stated clearly. However, we are not attempting to estimate the age of all users; we are only trying to estimate the age of a few users. This will allow us to perform validation for other, better-performing forms of age estimation. So, we choose to rely on some keywords that allow us to associate users with a notion of youth or old age. Here are the identified keywords.

Old : retraité, retraitée, senior, vieux, ex, exjournaliste, exprésident.

Young : étudiant, student, etudiant, étudiante, etudiante, jeune, junior.

We obtain an age for 2.2% of the users.

G.6 Keyword Gender

The last demographic information we try to estimate is gender. As for the age, this estimation will only serve us as validation, so we do not need to cover a large sample of users. In our dataset, very few users declare their pronouns, so it is not feasible to use this technique to estimate the gender of users. However, French (the predominant language in our dataset) is a gendered language, allowing us to identify the gender of the author (e.g., "je suis infirmier" indicates a male subject, while "je suis infirmière" indicates a female subject).

This technique, however, has two limitations: (1) there is not any consensual automatic model in French that can determine with certainty whether a word is feminine and whether it refers to the subject of the sentence; (2) if we identify feminine subjects, we cannot automatically ensure that the sentence is indeed self-declarative and refer to a person of feminine gender rather than another feminine entity (company, association, etc.). To address these issues, we have chosen to use the previously identified keywords to determine the gender of the users. Indeed, we have already taken care to determine that these keywords are self-referential, and in the case of occupation keywords, we know they refer to an individual rather than an entity. So we review the self-declarative keywords, and when possible, we note if these keywords are specifically masculine or specifically feminine. Here are some examples of identified keywords.

Masculine: président, conseiller, directeur, chef, passionné, consultant, citoyen, délégué...

Feminine: conseillère, directrice, ingénieure, présidente, experte, citoyenne, passionnée...

We notice above all that this method introduces a bias, as we can identify the gender only of users declaring an occupation or status. Additionally, we observe that in French, the masculine is considered neutral, so for certain occupations, we can only identify the feminine (e.g., "docteure" is feminine, but "docteur" can be used for both male and female), therefore, we have a slight selection bias towards women. Finally, given the unbalanced population, we expect to identify more men than women.

We obtain a gender for 20.5% of the users (14.5% men, 6% women).

G.7 Machine Learning Inference : Age, Gender and Organization

While keyword estimation for demographic features has the big advantage of being fast, highly auditable, and interpretable, they have some major drawbacks : (1) access to only a small sub-sample of users, (2) lack of representativity and biases (i.e., using occupation keywords for gender or age).

Fortunately, accurate machine learning models have been developed to tackle demographic inference on X. We use in our work the M3-inference model[35].

The M3-inference model takes as input Twitter profile pictures, screen names, and bios of users and uses a neural network multi-modal classification to predict :

- gender ('male' or 'female')
- age ('$\leq$ 18', '19-29', '30-39' or '$\geq$ 40')
- organization (i.e., if the profile belongs to an organization or a person: 'non-org' or 'is-org')

The model has been trained on heuristically identified and manually annotated data from multilingual users all over Europe. M3-inference has the particularity of using post-stratification to account for the biased samples of Twitter users, overperforming the state-of-the-art. On multilingual data, the F1-score performances are around 0.9 for gender and organization and 0.5 for ages.

We obtained on our dataset gender, organization status, and age for 89.5% of the users (72.1% of males and 27.9% of females among "non-organization" profiles).

We compared the result of the M3-inference with the keyword inference presented before. We see figure S32 that keyword and M3-inference are coherent in identifying genders, ages, and organizations. For gender and organization, the keyword methodology reached F1 scores near the base performance of the M3 methodology. This is an important validation for the keyword methodology in general.

For ages, the coherence has been lower due to the imprecision of the two measures. In particular, M3-inference struggles to identify ages without uncertainty, and most of the predictions do not return a clear majority for one class but instead nuanced probabilistic results such as: `{'<=18': 0.2712, '19-29': 0.6259, '30-39': 0.0948, '>=40': 0.0082}`. To take into account the uncertainty we define a new value called "estimated age" which is calculated by attributing an age for each age category and then computing the expected age according to the mass probability distribution.

H Statistical dependence among demographic features

Collecting demographic data from users is crucial for two main reasons. Firstly, it enables us to broaden our explanatory method beyond simple political data. Secondly, it allows us to identify potential covariates while studying the relationships between political positions and latent space.

Indeed, we expect, from the literature, users' political positions to be linked to socio-demographic features. Socio-demographic features related to political positions and sharing behaviors could ultimately be covariates in our explanation process (i.e., if we find a latent dimension of the algorithm carrying political information, it could simply measure the age of users, subsequently correlated with political positions). Thus, to spot potential covariates, we need to highlight the statistical relation between demographic variables and attitudinal ones.

In this section, we first examine the correlations among our socio-demographic variables, observing the co-occurrence of occupation, qualifiers, and topics in our dataset. We then examine the political positions of different populations based on our five socio-demographic variables: occupations, statuses, topics, gender, and age. We aim to understand the impact of these variables on political positions and explore any significant differences between populations.

H.1 Co-occurence of occupations, qualifiers and topics

As each user can declare multiple occupations, statuses, and topics, we can measure their co-occurrence in our dataset.

We measure the statistical dependency between each pair of socio-demographic keyword features (occupation, qualifier, or topic) s_i and s_j. We calculate the conditional probabilities $P(s_i|s_j)$ and $P(s_j|s_i)$ to measure the size effect of the dependence. We use the Chi-squared test of independence to assess the statistical significance of the relation.

Considering only the relations where $P(s_i|s_j) \geq 0.15$ and $p_{\text{value}} < 10^{-5}$, we can build a directed graph representing which socio-demographic keywords are co-occurring.[27] We represented this graph figure S33, showing only links with $P(s_i|s_j) \geq 0.15$ for clarity of the visualization.

Observing the figure S33, we notice different groups of co-occurring keywords. We can precisely identify those groups by using the Louvain community detection method[28][36]. We used the Louvain community detection on the undirected weighted graph using as weights $w_{ab} = P(A|B) + P(B|A)$. We obtained several empirical groups of co-occurring keywords that we distinguished by the shape of nodes in our graph.

Table S3 we present the main co-occurrence groups obtained by performing Louvain community detection on the graph of dependencies, considering as link all the dependencies with $P(s_i|s_j) \geq 0.01$ and $p_{\text{value}} < 10^{-5}$. For each group, we notice common domains of interest, and we can then perform post hoc interpretations to understand the reasons for co-occurrences.

Before analyzing the result of this community detection, we want to emphasize that these groups are groups of shared declared identity and not networks of relations. Having analysts and consultants in the same group of co-occurrences does not mean that analysts are highly connected to consultants or that they form a community in the real world; it means that a significant number of users declare to be both analysts and consultants. We find this metric particularly interesting because it is rare in the academic literature on social media, which often focuses on links and communities rather than identity. Especially in light of recent works showing the importance of identity in public activities online (such as link sharing)[37].

Identifying empirical groups of co-occurring keywords is particularly useful as validation or rejection for our theoretical groups of occupations presented table S2. In fact, our four main empirical groups (Politics, Academia, Business, and Journalism) match pretty well with the four socio-professional categories proposed by the CSP classification identified in our dataset.

Nonetheless, this technique also allows us to identify differences between theoretical and empirical classification for our data.

[27] Note that we consider only p_{value} particularly low because we have 167 different socio-demographic keywords we are hence testing for $167^2 \approx 10^4$ relations, usual p values of order 10^{-3} would result in an important number of false positives.

[28] based on the maximization of modularity between clusters

- We identified a group of co-occurring keywords around the ecological topics (previously invisible using only the CSP classification), containing topics such as climate and biodiversity that extend to all the main sectors related to climate transformation, such as construction, energy, agriculture, mobility... This new group informs us on the importance of this emerging interdisciplinary field in displaying online identity and gives us a hint on the kind of actor qualifiers linked to the ecological topic: i.e., activist and citizen.
- Developers and engineer occupations, which are socio-professionally classified in "Business, IT and administration professionals", are empirically more used with identity linked to academia.

Finally, this validation allows us to identify some anomalies that illuminate the limits of theoretical classification based on keywords.

- Farmers are linked to journalism because of the ambiguity of the keyword "producer"; in fact, while most of the producers are actual audiovisual producers, a significant part of farmers also use the word producer, thus creating an asymmetric conditional link from farmer to producer.
- The qualifier "professional" is used by a large group of different occupations, but it is more common for certain specific occupations, such as firefighters.

We choose for our analysis to consider these groups as **declared interests** of users. This denomination is vague on purpose because the extent of our work does not give us the time to investigate further the link between identities and auto-declarative keywords used, while only hypothesizing an interest of the user in the particular domain requires less proof. However, we keep in mind that this specific partition of the interest is related to the keyword used by users to describe themselves, and the interpretations made are post-hoc ones with the goal of simplification for the reader. We do not prove at any moment that the interest linking all the users in a specific group is, in fact, the topic we proposed as the name.

H.2 Relationships between political positions and Demographic Features

There exists extensive literature linking political opinions and socio-demographic characteristics[38,39]. In this section, we specifically explore this connection within our dataset.

Statistically, our goal is to describe the relationship between a set of categorical variables (socio-demographic features) and two continuous variables (political positions). We measure (1) the existence of an effect (using a Welch t-test) and (2) the effect size (measuring the mean difference by group). The Welch t-test is based on the hypothesis that political positions follow a normal distribution, which is the case in our dataset.[29]

In the following analysis, we consider each class of each socio-demographic feature (e.g.: age ≤ 18, gender male...) as a Bernoulli variable A. For each couple of socio-demographic variables and political positions, we conducted a Welch t-test to test the dependence between the two variables. When a significant dependence is detected, we report the difference in the average political attitude between individuals who belong to the specific socio-demographic class (i.e., $A = 1$) and those who do not (i.e., $A = 0$). We finally calculate the confidence interval for this effect, depending on the uncertainty in estimating the means.

The Welch t-test is a variant of Student's t-test that tests the null hypothesis that two normal distributions (of different variances) have the same mean. In our case for a binary sociodemographic variable s_i and a political variable ϕ we test the hypothesis that the mean of ϕ knowing s_i ($\mu_{\phi|s_i}$), equal the mean of ϕ knowing $\neg s_i$ ($\mu_{\phi|\neg s_i}$), under the assumption that $\phi|s_i$ and $\phi|\neg s_i \sim \mathscr{N}$ (which was tested before). The significance levels are indicated by asterisks in the figures, with $p_{\text{value}} < 10^{-2}$ denoted by *, $p_{\text{value}} < 10^{-3}$ by **, and $p_{\text{value}} < 10^{-4}$ by ***.

The confidence interval for the mean difference is equal to the sum of the confidence intervals for the estimation of both means. It was calculated using the standard formula for confidence intervals in mean estimation for normal distributions:

$$\text{Confidence Interval} = \mu \pm z \times \left(\frac{\sigma}{\sqrt{n}}\right)$$

Where μ is the sample mean, σ is the sample standard deviation, n is the sample size, and z is the critical value of the standard normal distribution corresponding to the desired confidence level (in our case, we used a 99% bidirectional confidence interval to match our p value of 10^{-2}).

We then get the group mean difference with confidence interval for a socio-demographic feature s_i and a political attitude ϕ as :

[29] While we don't theoretically expect the political positions to follow normal distributions, standard tests of normality failed to reject the null hypothesis that political positions follow a normal distribution (e.g., Shapiro test $p_{\text{value}} \approx 1.0$). We then consider the attitude distribution close enough to the normal distribution to make the test valid.

$$\text{GMD}(s_i,\phi) = \mu_{\phi|s_i} - \mu_{\phi|\neg s_i} \pm 1.96\left(\frac{\sigma_{\phi|s_i}}{\sqrt{|\mathscr{U}_{s_i}|}} + \frac{\sigma_{\phi|\neg s_i}}{\sqrt{|\mathscr{U}_{\neg s_i}|}}\right)$$

Figure S19 we see the mean difference in political attitude for each demographic group. Females are, on average, slightly Left-leaning, which is coherent with the already documented gender gap in political positions[40]. As expected, organizations' accounts tend to be significantly less anti-elite than personal accounts, since organizations often represent the institutions themselves. Age seems to have a significant impact on anti-elite salience. These results seem coherent to the French case where, for example, parties displaying anti-elite stances (Rassemblement National or La France Insoumise) receive more votes from young voters compared to low anti-elite parties (Renaissance) receiving more votes from older voters[41]. However, works in other European countries show opposite results, which nuance this relation[42]. In particular, we think it is probable that the observed effect in our dataset is in part due to a selection bias on X. In fact, because Twitter is biased toward high SES users, because age is positively related to higher wages and socioeconomic status[30] and because high SES is related to lower anti-elite sentiment, then we could expect old users on Twitter to show lower anti-elite sentiment with SES as a covariate.

In Figure S25, we see the mean difference in political attitude for each occupation group, and in Figure S28, each declared interest group. The mere presence of a declared occupation or interest in user bios generally correlates with lower Anti-elite attitudes. This is likely because socio-professional categories are self-reported, and users with higher socioeconomic status are more inclined to disclose their occupations and qualifications publicly online than anti-establishment ones. We notice a general coherence in the political positions of corresponding socio-professional and declared interest groups (in part because they are measured using the same keywords).

Most of the groups are independent of Left-Right ideology, except for business, which tends to be right-leaning in interest and occupation, and ecology, which is left-leaning. This is coherent with French right-wing political parties usually defending liberalism and businesses, and left-wing parties promoting ecological policies.

Finally, as anticipated, political occupations, which are inherently associated with the establishment, exhibit significantly lower Anti-elite attitudes compared to all other professions.

It is also interesting to notice that while professors and researchers on Twitter seem to be, on average, left-leaning, the whole of academia (which includes a greater variety of users related to knowledge, such as think tanks and alumni) seems more balanced.

In this section, we mainly focus on the fact that some of the socio-demographic features we estimated are statistically related to political positions and can then be considered potential covariates. These factors need then to be disentangled from political positions in the explanation process.

I URLs belonging to known medias

We identified known French media among the domains in our training set. We took as reference an extensive study presenting an overview of the French media ecosystem[43]. This study built a dataset with 478 primary French media online and studied the citation network among those media. From the citation network, groups were identified resulting from Stochastic Block Model community detection[44]. Analysis of those citation groups allows the building of a classification of the media. We presented the classification tab S4. The classes have high inner group citations and low outer group citations; the names given are the result of the interpretation proposed by Cointet et al. In addition to the classification, the dataset gives, for each media, information on the type of media (s.a., their outreach, their editorial structure, or their origin).

We matched our collected URLs with media when possible. Each media outlet can be represented by multiple URLs (e.g., Le Monde can be lemonde.fr, blog.lemonde.fr...). In our dataset, we were able to recognize 1024 URLs belonging to 440 different media; we call those media "recognized media."

We observed in both our latent space and in our input data that URLs from the same media are, on average, more similar to each other than random URLs. Furthermore, the number of interactions (i.e., sharing from Twitter users) for each media is linearly correlated with the number of citations that this media gets from other media. This validates our method of considering media belonging as useful representations of URL features.

J Socio-demographic explanation

While dimensions L_3 and L_4 encode in the ideological features of individuals and recommendable contents, an important question is whether they *also* encode additional relevant features that might be highly correlated with ideology. In the most extreme case, if ideology is completely correlated with, say, age and gender, the recommender might learn dimensions for all these features jointly, in which case it would be meaningless to distinguish a political dimension. To assess the independence

[30] This is the case in our dataset

of learned political representations, we control for natural covariates that might be correlated with ideology: age, gender, occupation, language, and declared interest.

The covariates considered are :

- demographic features: gender, age (estimation section G.7), and language (section G.1)
- socio-demographic features: occupation group (section G.2) and declared interest (section H)

Since these features primarily concern personal users, we remove profiles belonging to organizations from this section. We saw section H.2 that those features are statistically related to political positions, and that they can then behave as proxies of the political positions. Finally, we considered the media category (section I) of each recognized URL not as covariates, but as validation, since among those categories, some bring inherent information on the political leaning of the media (Left-wing, Right-wing, Identitarian and Revolutionary Right).

To isolate the variance uniquely driven by political positions from that captured by their socio-demographic proxies, we compute (for each latent dimension of our embedding) the local discrete attribution factor of each class of socio-demographic feature, using the method of proxy attribution presented in section F. We expect dimensions learning political positions solely to attribute high impact to political media but low impact to socio-demographic features. We expect dimensions learning political positions as proxies of socio-demographic to attribute moderate impact to political media, while attributing high impact to socio-demographic features. Finally, we expect some dimensions to learn exclusively socio-demographic features without political positions simply due to the importance of those features on media consumption. While observing a dimension attributing impact both to political and demographic features won't prove that one is the proxy of the other, observing a dimension attributing impact to one and not the other will prove that no variable is a proxy of the other.

From figure S34 to figure S45, we plot for each latent dimension the six highest positive and six lowest negative attribution values of socio-demographic classes. Attribution values are expressed in units of the standard deviation of the respective latent dimension and highlight the classes exerting the strongest directional influence. The dashed vertical line marks and the zero-attribution baseline are used solely for visual alignment and comparison between panels; they do not represent a statistical significance threshold.

The first results to notice figure S37 and figure S38 is that socio-demographic proxies (age, gender, occupation, language, and declared interest) exert minimal influence on latent dimensions L_3 and L_4. These findings suggest that the model captures political positions largely independently of users' demographic characteristics. This observation is further reinforced by the fact that media leaning shows a much stronger explanatory relationship with these dimensions.

The second result is that some latent dimensions show substantial alignment with demographic features. For example, latent dimension L_1, figure S35, can predominantly be associated with users in business and IT (simultaneously for occupations, interest, and media). These findings confirm that the model is capable of learning demographic representations when they are relevant. The lack of demographic signal in L_3 and L_4, therefore, reflects a functional specialization of these dimensions toward ideological encoding, rather than a general insensitivity of the model to demographic information.

While other untested features may be at stake, one can attempt to interpret the result by examining the tendencies in the impact of each class. Here are the tendencies identified :

- L_0 : local – French
- L_1 : Business and IT
- L_2 : international
- L_3 : Right wing
- L_4 : Left wing
- L_6 : local – regional
- L_{11} : Arabic content

To overcome potential limitations of discrete attribution factors as an explanation tool, these results have been replicated using other indicators, such as ROC-AUC, and show similar results.

Figures and Tables

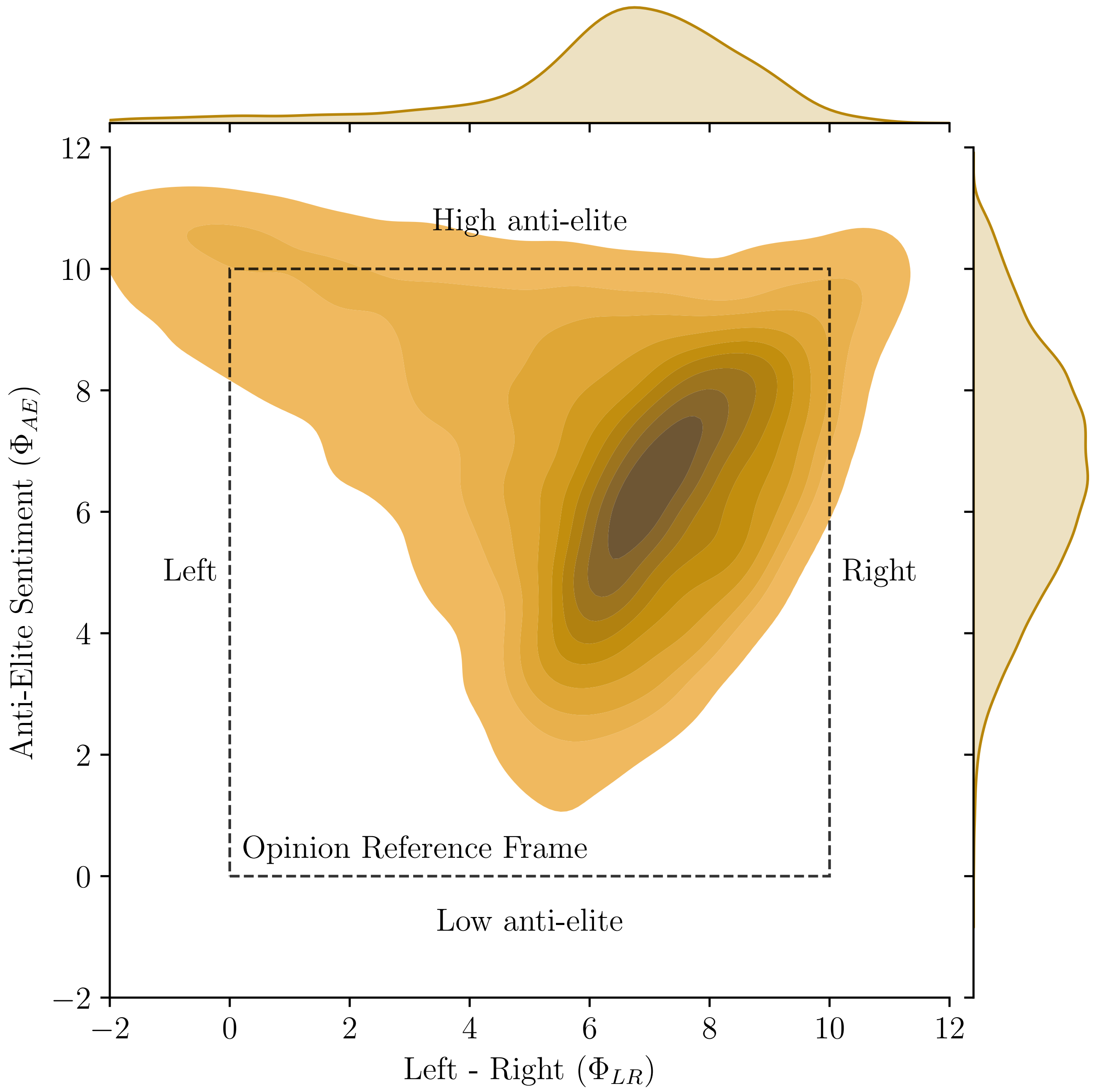

Figure S1. Spatial distribution of the user dataset on the two main political dimensions of French political spectrum : Left-Right and Anti-elite sentiment (CHES dimensions).

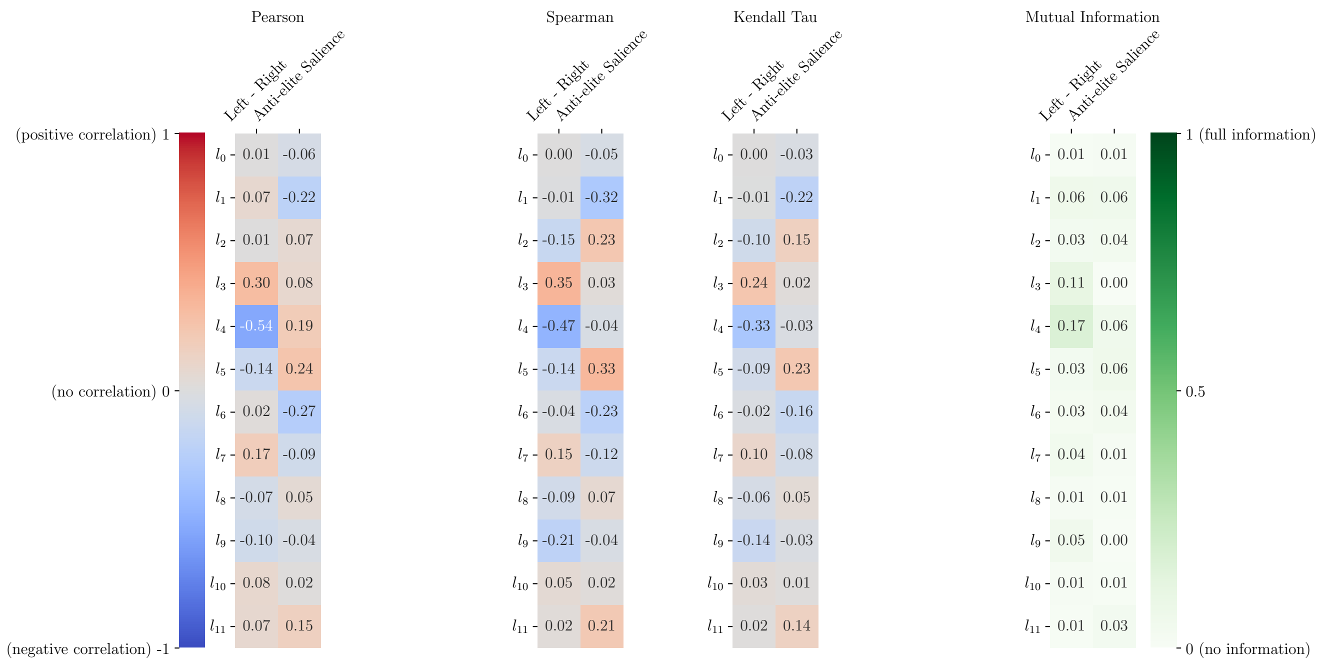

Figure S2. Statistical correlations between latent dimensions and political dimensions of users with standard indicators are coherent with the explanation indicators.

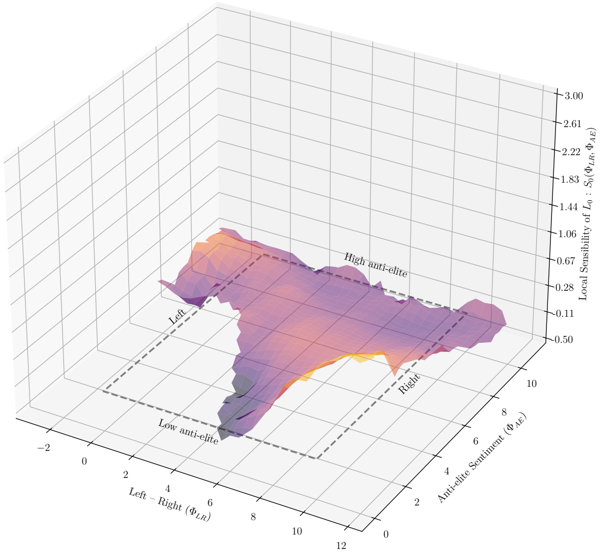

Figure S3. Local attribution factor of L_0 to political positions (no effect).

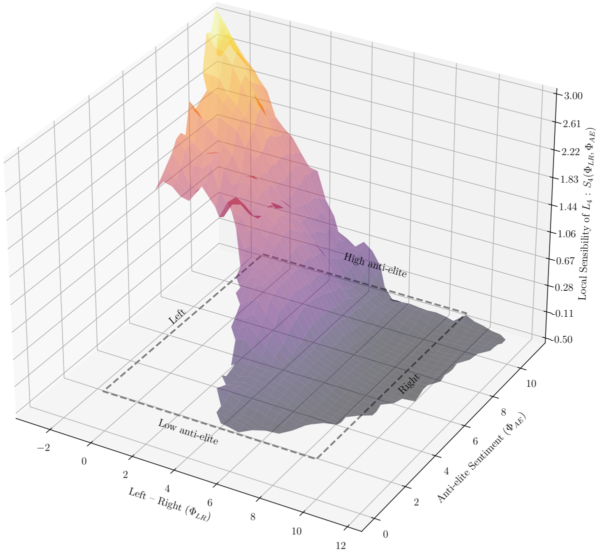

Figure S4. Local attribution factor of L_4 to political positions (sensible to Left ideology and Anti-elite attitudes).

Figure S5. The local attribution factor of L_0 and L_4, measured as $S_j(\phi_{LR}, \phi_{AE}) = \frac{\mathbb{E}[L_j|(\Phi_{LR}, \Phi_{AE})] - \mathbb{E}[L_j]}{\sigma_j}$. On the left, we observe $S_0(\phi_{LR}, \phi_{AE}) \approx 0$ indicating that L_0 is not sensible to Φ_{LR}, Φ_{AE} variations. On the right, we see that L_4 reacts to the Left ideology and Anti-elite attitudes.

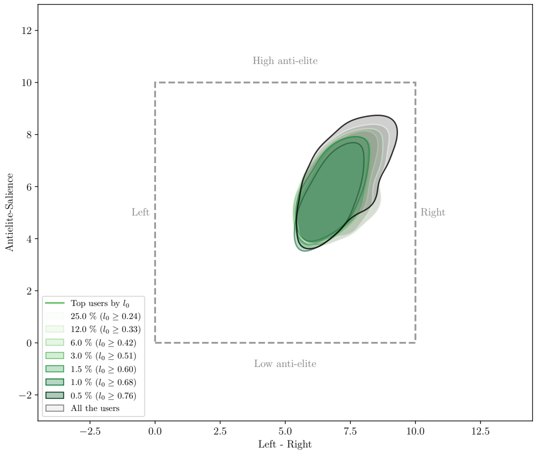

Figure S6. Political latent Leaning and Diversity for top L_0 users (no effect).

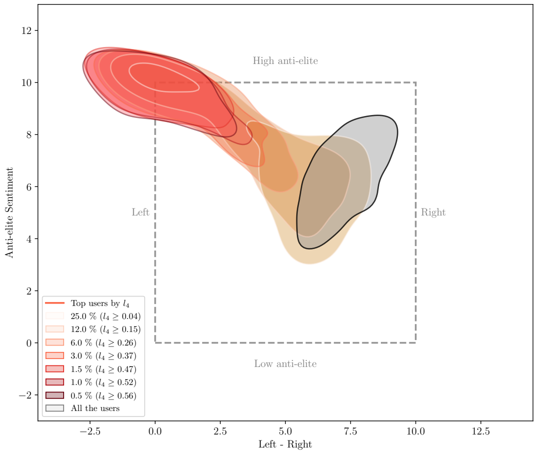

Figure S7. Political latent Leaning and Diversity for top L_4 users (Significant leaning toward Left, Anti-elite).

Figure S8. We compare the political latent Leaning and Diversity for L_0 (no effect) and L_4 (leaning toward Left, Anti-elite). We plot the conditional distribution of political positions of the top users for latent dimensions 0 (L_0 in green) and 4 (L_4 in blue); we plot the distribution of random users in black as reference. The leaning is the distance of the conditional distribution (color) from the random one (black), and the latent diversity is the width of the conditional distribution compared to the random one. The political position distribution of users with high L_0 are similar to random users, but the political positions of users with high l_4 are significantly different than the distribution of average users; we observe a leaning toward Left, Anti-elite attitudes.

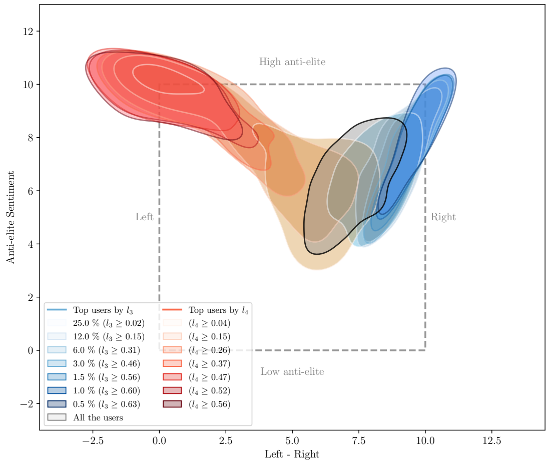

Figure S9. Political latent leaning and diversity for the two dimensions with the highest attribution factor for Left-Right ideology (L_3, L_4)

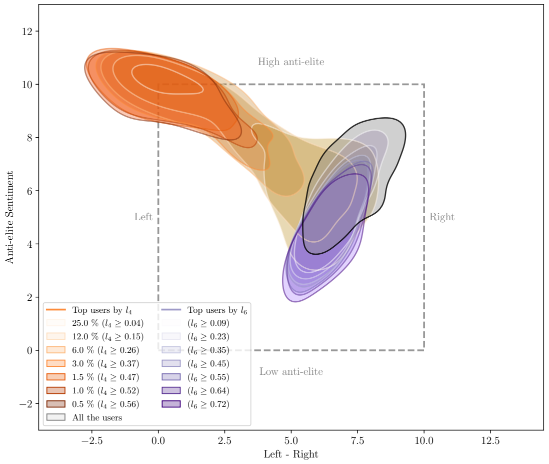

Figure S10. Political latent leaning and diversity for the two dimensions with the highest attribution factor for Anti-elite sentiment (L_4, L_6)

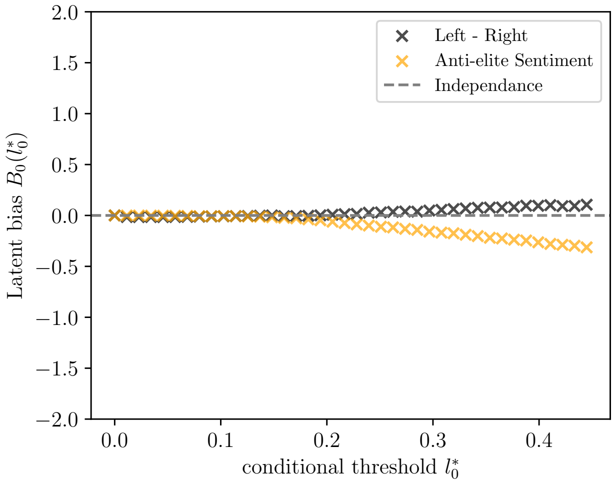

Figure S11. Political latent Leaning of L_0 (no leaning).

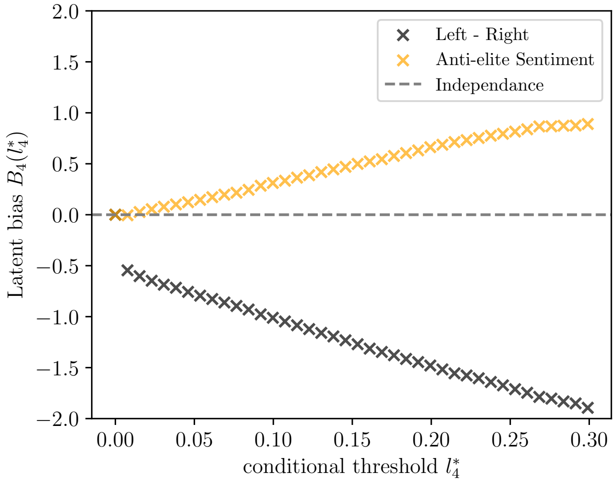

Figure S12. Political latent Leaning of L_4 (Left and Anti-elite leaning).

Figure S13. The political position latent leaning of L_8 and L_4, measured as $B_{j,\Phi}(l_j^*) = \frac{\mathbb{E}_\phi[\Phi|(L_j > l_j^*)] - \mathbb{E}_\phi[\Phi]}{\sigma_\Phi}$. Measure how the top users from one dimension are politically different from random users. The direction of the leaning determines the sign.

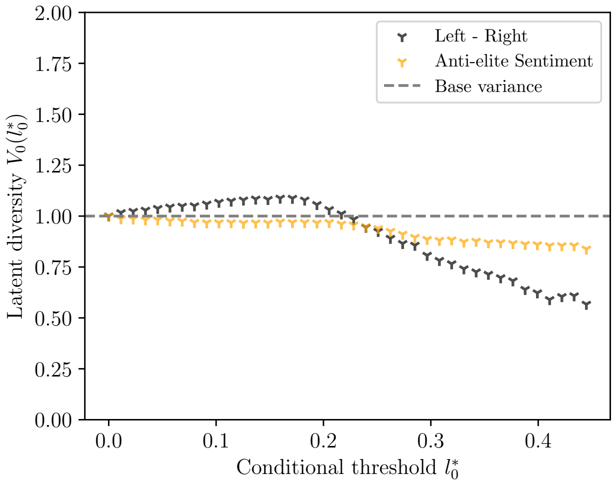

Figure S14. Political latent diversity of L_0 (Low diversity: specializing in hyper-center users).

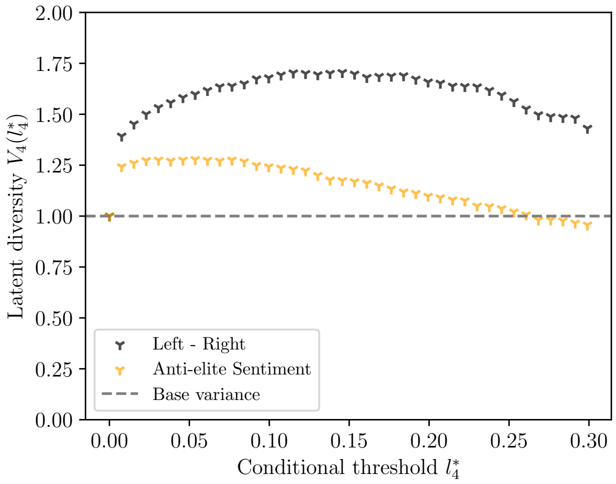

Figure S15. Political latent diversity of L_4 (politically diverse: including both Left and Far-Left users).

Figure S16. The political position latent diversity of L_0 and L_4, measured as $V_{j,\Phi} = \frac{\mathrm{Var}(\Phi|L_j > l_j^*)}{\mathrm{Var}(\Phi)}$. Measure how the top users from one dimension are politically diverse.

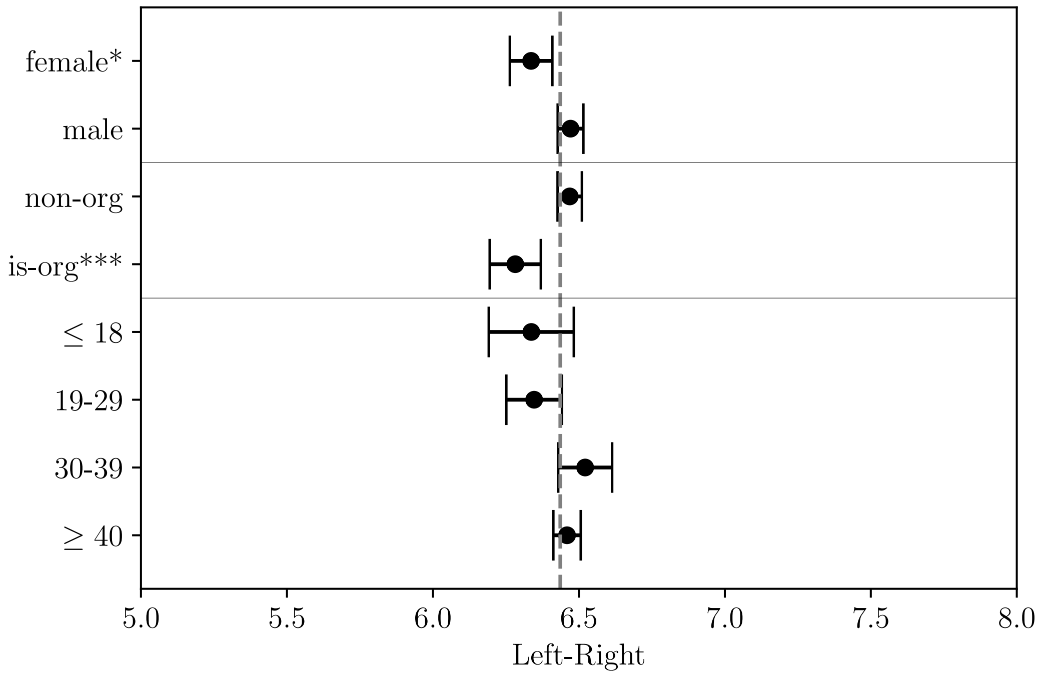

Figure S17. Demographic features (LR)

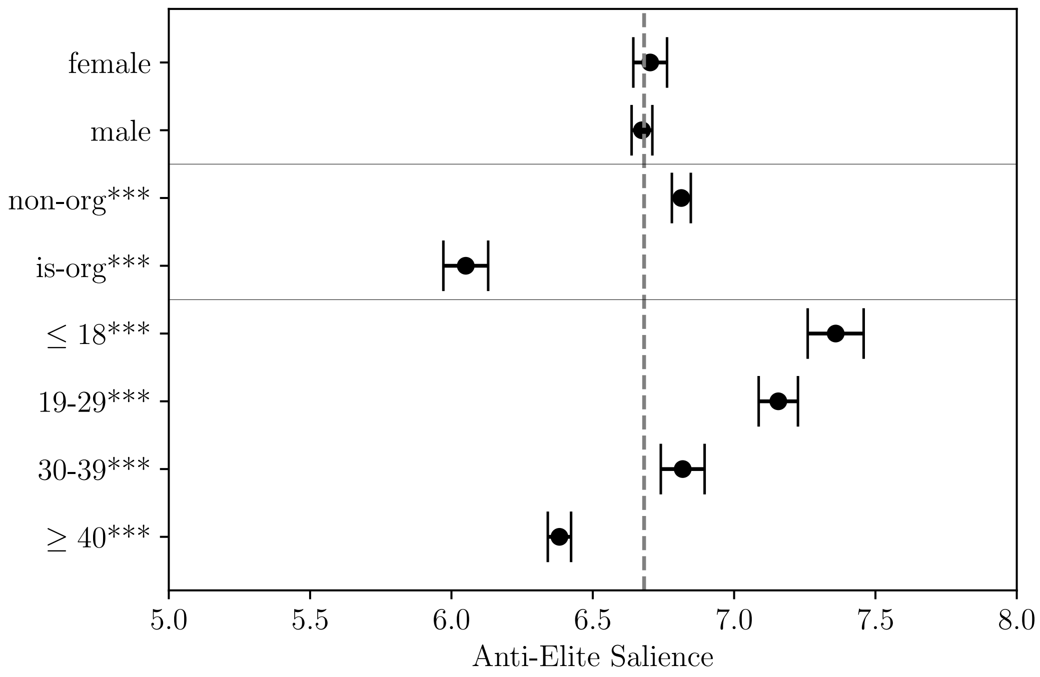

Figure S18. Demographic features (Anti-elite)

Figure S19. Average political position depending on **demographic features**. In gray is the overall mean; error bars represent the uncertainty on the sample mean. The asterisks indicate the statistical significance of the mean difference: * $p_{\text{value}} < 10^{-2}$, ** $p_{\text{value}} < 10^{-3}$, *** $p_{\text{value}} < 10^{-4}$.

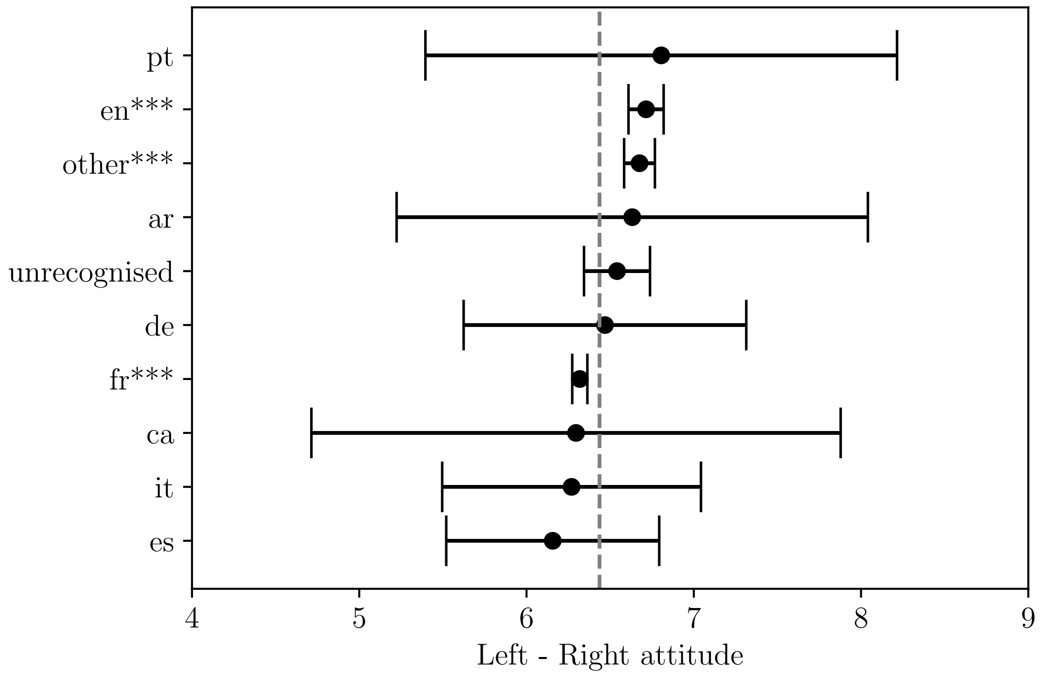

Figure S20. Language (LR)

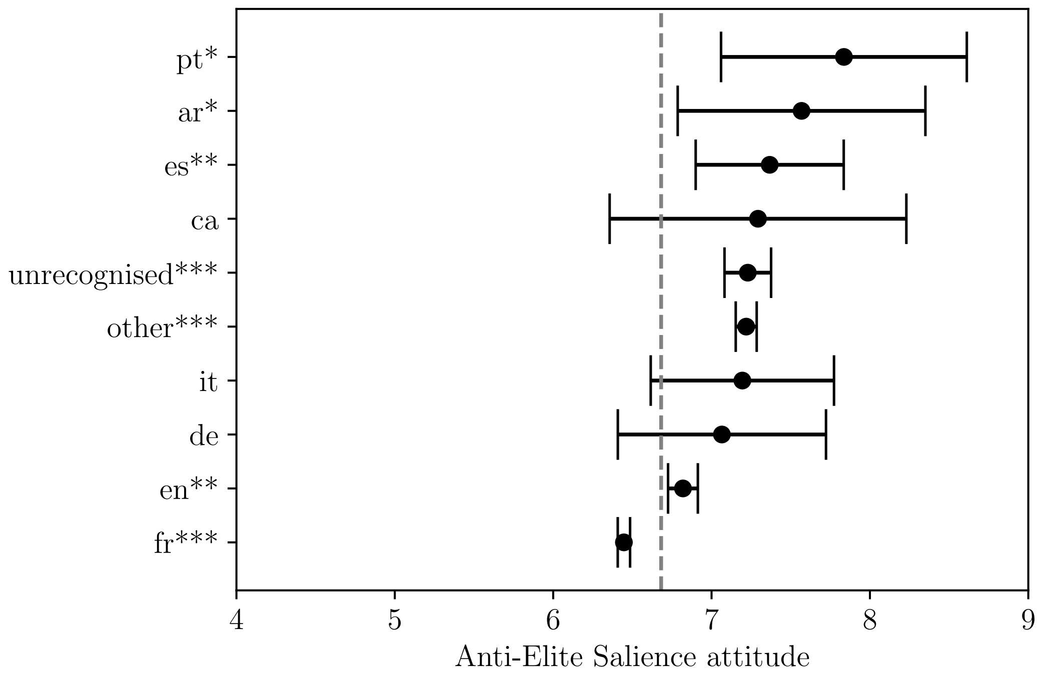

Figure S21. Language (Anti-elite)

Figure S22. Average political position depending on **language**. In gray, the overall mean; error bars represent the uncertainty on the sample mean. The asterisks indicate the statistical significance of the mean difference: * $p_{\text{value}} < 10^{-2}$, ** $p_{\text{value}} < 10^{-3}$, *** $p_{\text{value}} < 10^{-4}$.

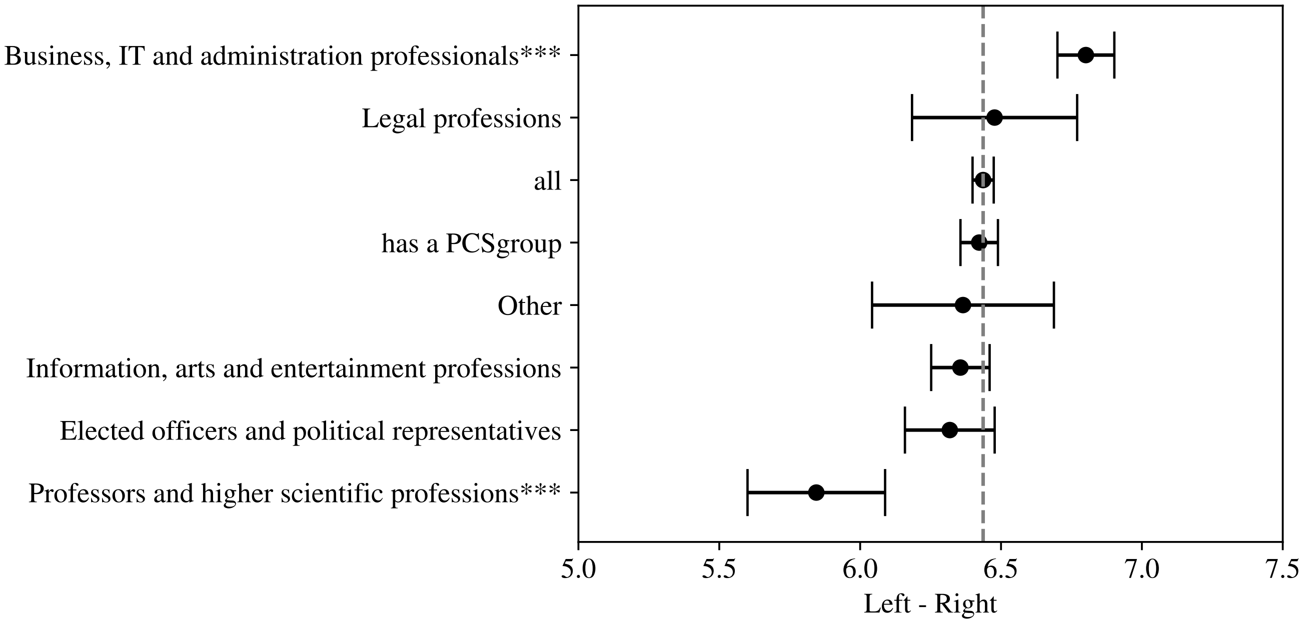

Figure S23. Socio-professional categories (LR)

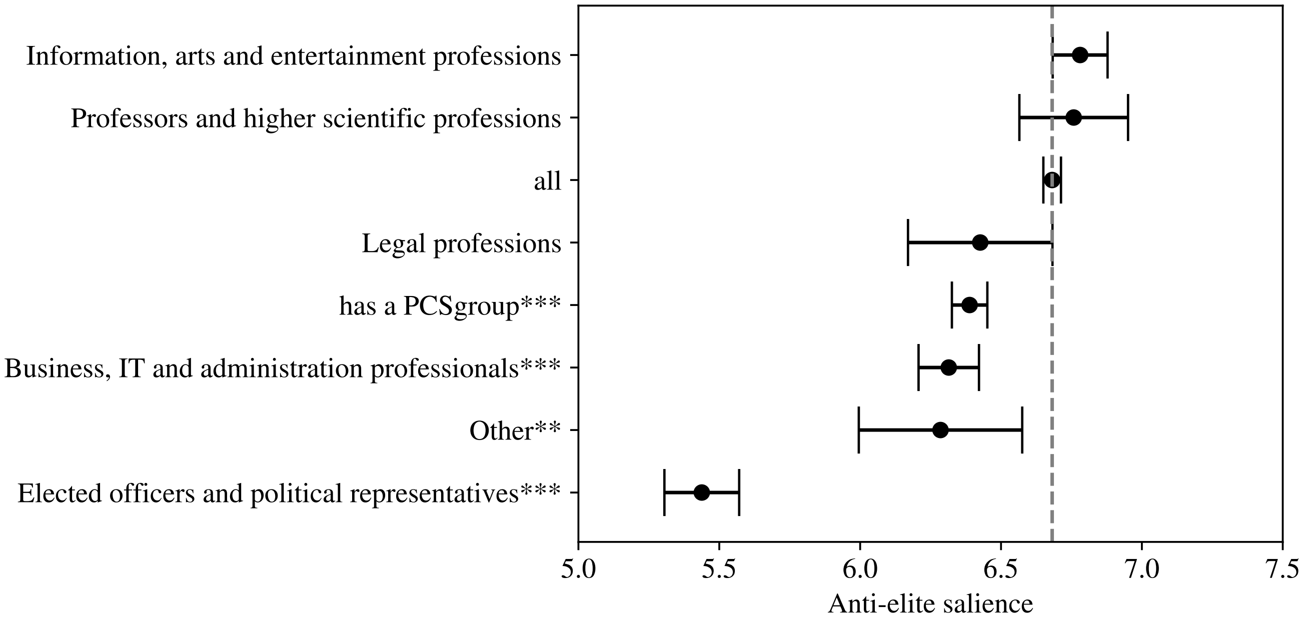

Figure S24. Socio-professional categories (Anti-elite)

Figure S25. Average political position depending on **socio-professional categories**. In gray, the overall mean; error bars represent the uncertainty on the sample mean. The asterisks indicate the statistical significance of the mean difference: * $p_{\text{value}} < 10^{-2}$, ** $p_{\text{value}} < 10^{-3}$, *** $p_{\text{value}} < 10^{-4}$.

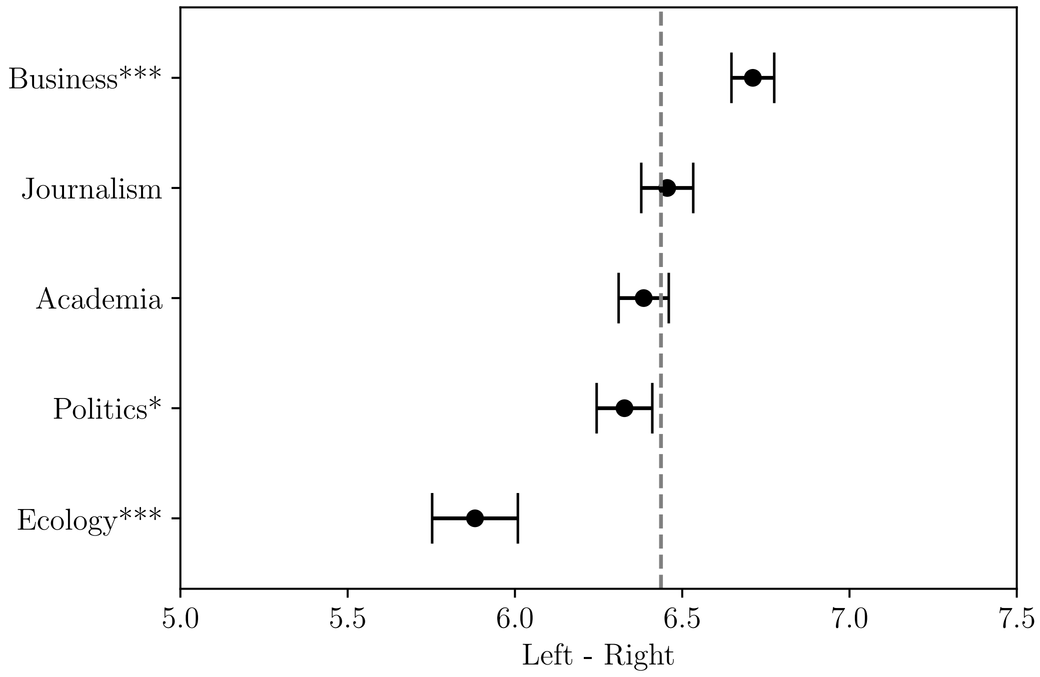

Figure S26. Declared interest groups (LR)

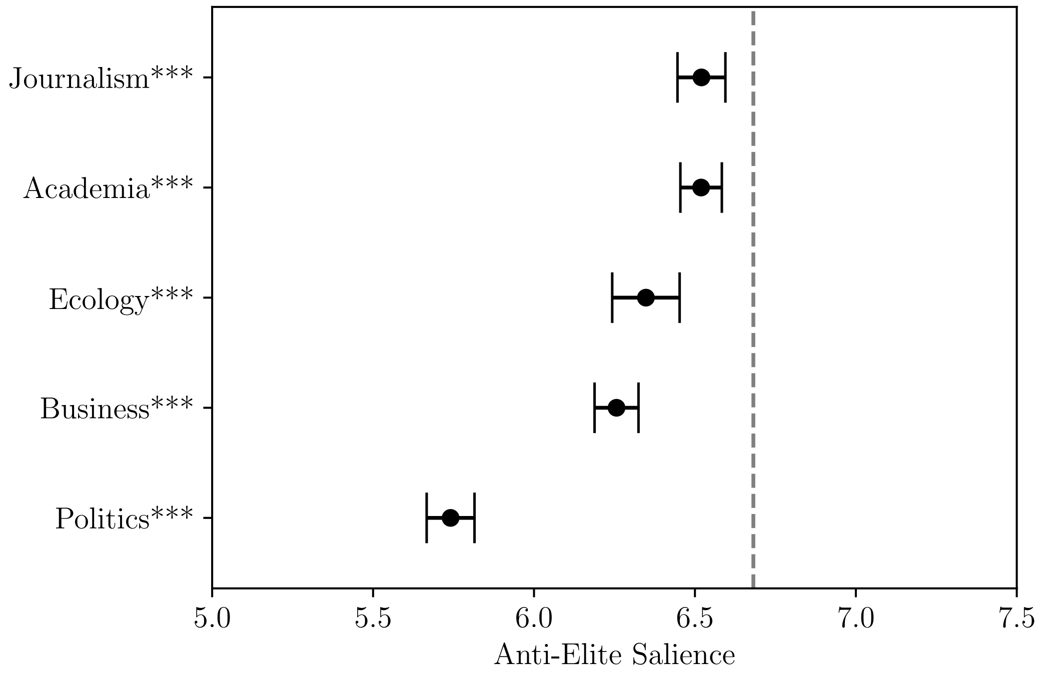

Figure S27. Declared interest groups (Anti-elite)

Figure S28. Average political position depending on **declared social group**. In gray, the overall mean; error bars represent the uncertainty on the sample mean. The asterisks indicate the statistical significance of the mean difference: * $p_{\text{value}} < 10^{-2}$, ** $p_{\text{value}} < 10^{-3}$, *** $p_{\text{value}} < 10^{-4}$.

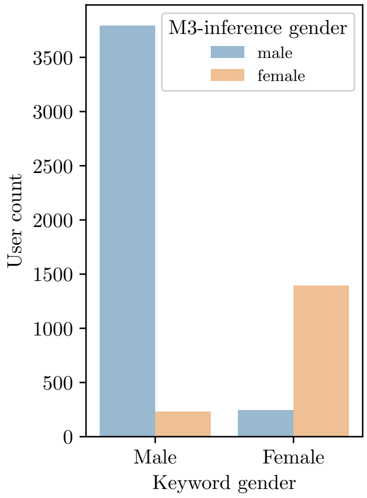

Figure S29. Gender: $F1 = 0.94$

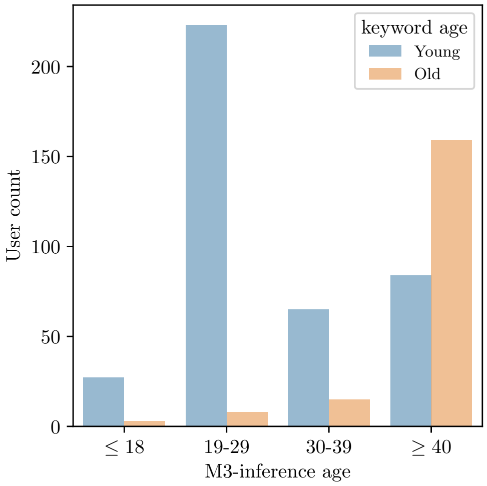

Figure S30. Age

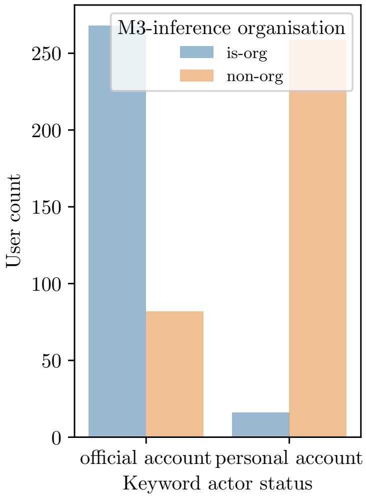

Figure S31. Organisation: $F1 = 0.84$

Figure S32. Comparison between M3-inference and keyword methodology to infer demographic features (among users classified by keyword). We see that the keyword methodology is coherent with the ML inference.

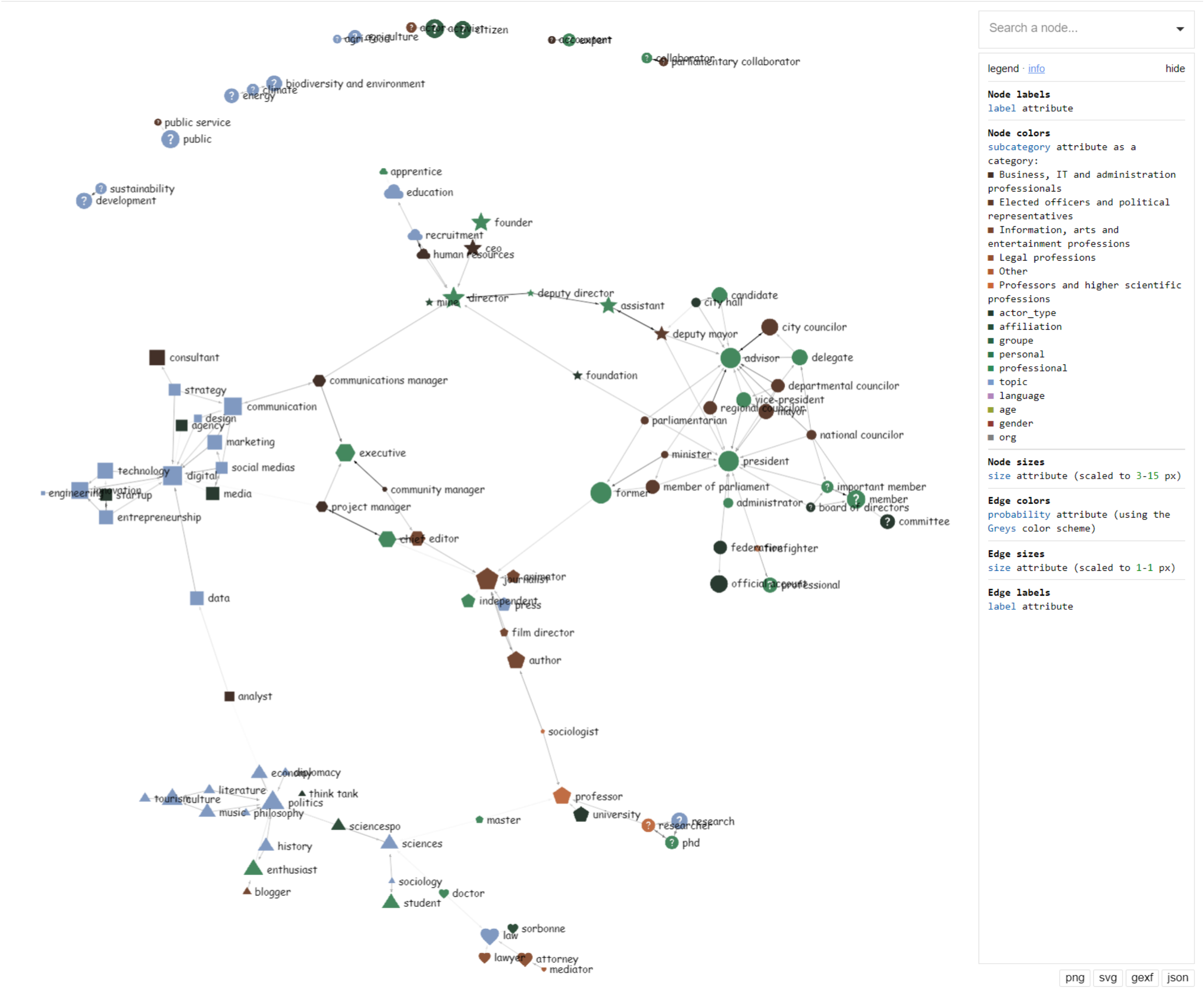

Figure S33. The directed graph representation of the dependence among occupation, qualifier and topic keywords. The intensity of the links represent the conditional probability of the destination keyword knowing the source keyword. The shape of the nodes represent the groups of co-occurring keywords detected on the graph by Louvain method. The color of the node indicate the type of keyword (Occupation, Qualifier, Topic...).

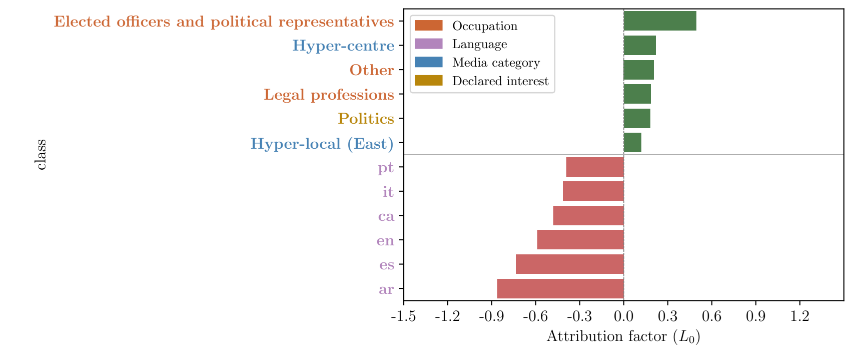

Figure S34. Socio-demographic features with the highest impact on latent dimension L_0 (tendency: local - French).

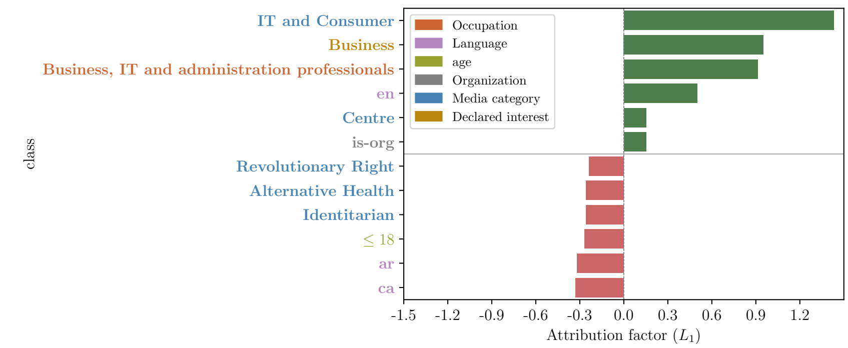

Figure S35. Socio-demographic features with the highest impact on latent dimension L_1 (tendency: Business and IT).

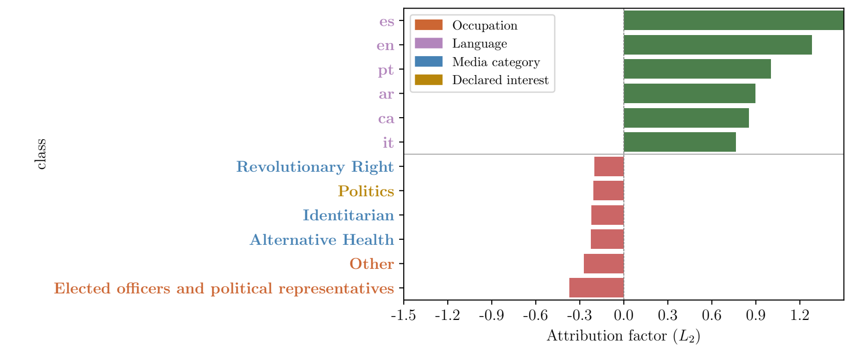

Figure S36. Socio-demographic features with the highest impact on latent dimension L_2 (tendency: international).

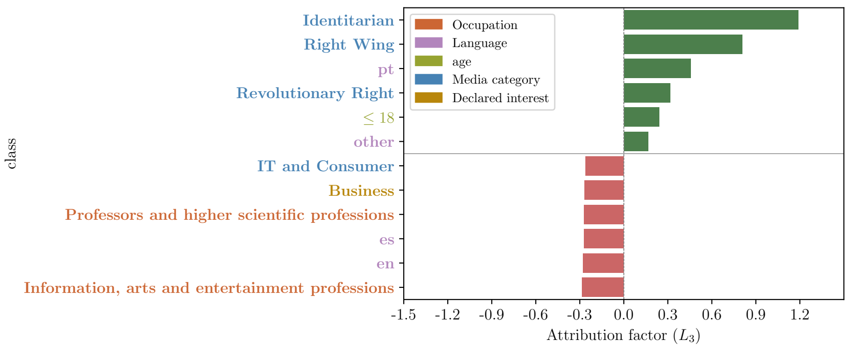

Figure S37. Socio-demographic features with the highest impact on latent dimension L_3 (tendency: Right wing).

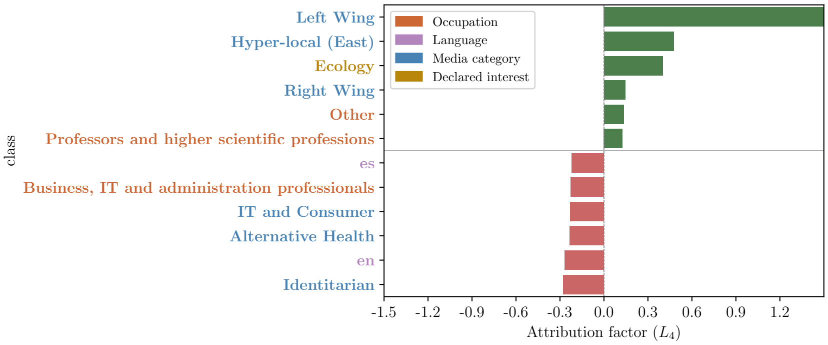

Figure S38. Socio-demographic features with the highest impact on latent dimension L_4 (tendency: Left wing).

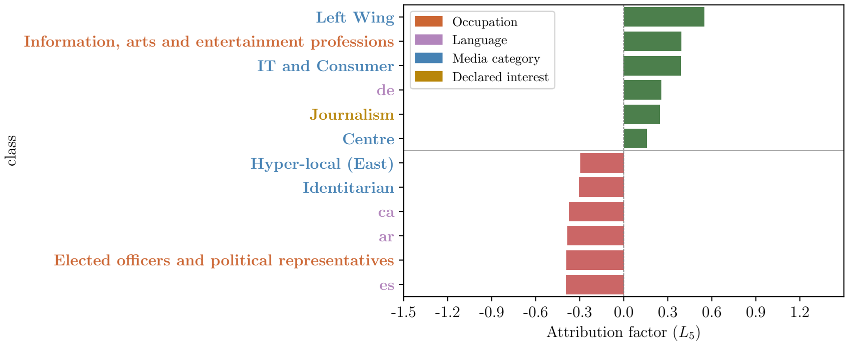

Figure S39. Socio-demographic features with the highest impact on latent dimension L_5.

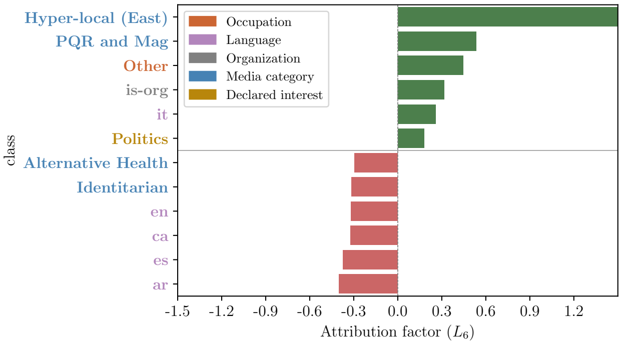

Figure S40. Socio-demographic features with the highest impact on latent dimension L_6 (tendency : local - regional).

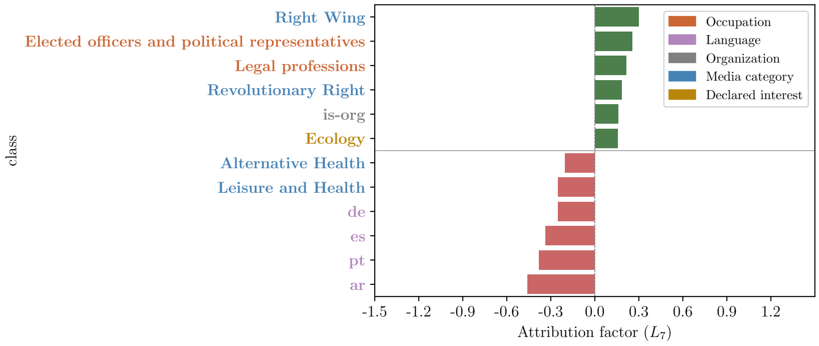

Figure S41. Socio-demographic features with the highest impact on latent dimension L_7.

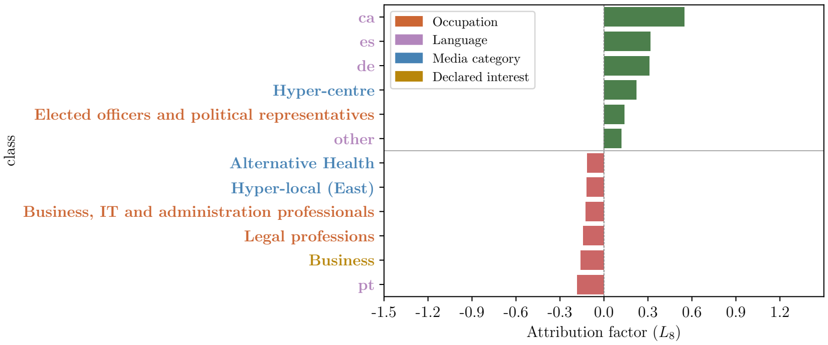

Figure S42. Socio-demographic features with the highest impact on latent dimension L_8.

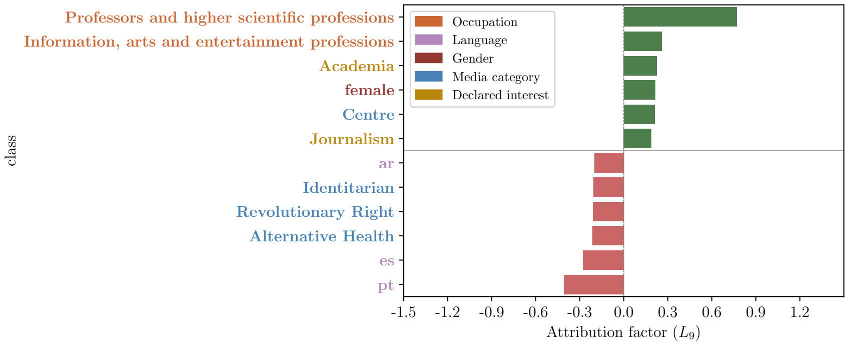

Figure S43. Socio-demographic features with the highest impact on latent dimension L_9.

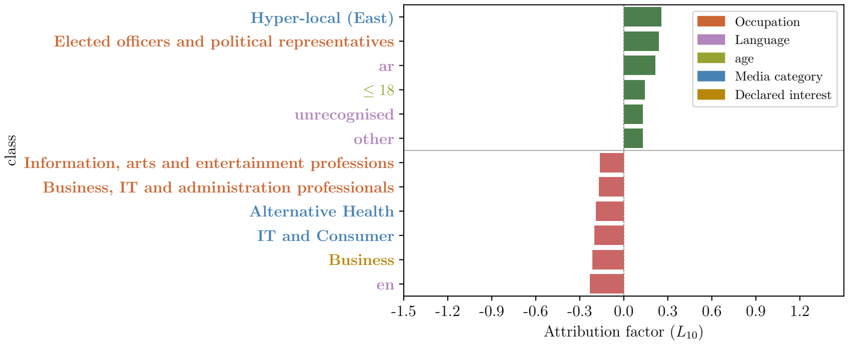

Figure S44. Socio-demographic features with the highest impact on latent dimension L_{10}.

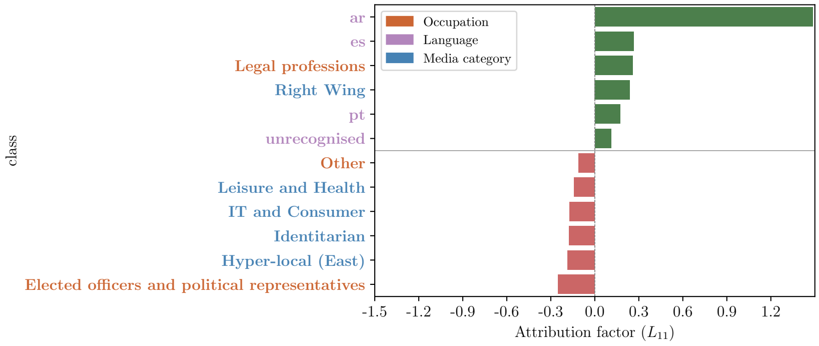

Figure S45. Socio-demographic features with the highest impact on latent dimension L_{11} (Tendency : Arabic content).

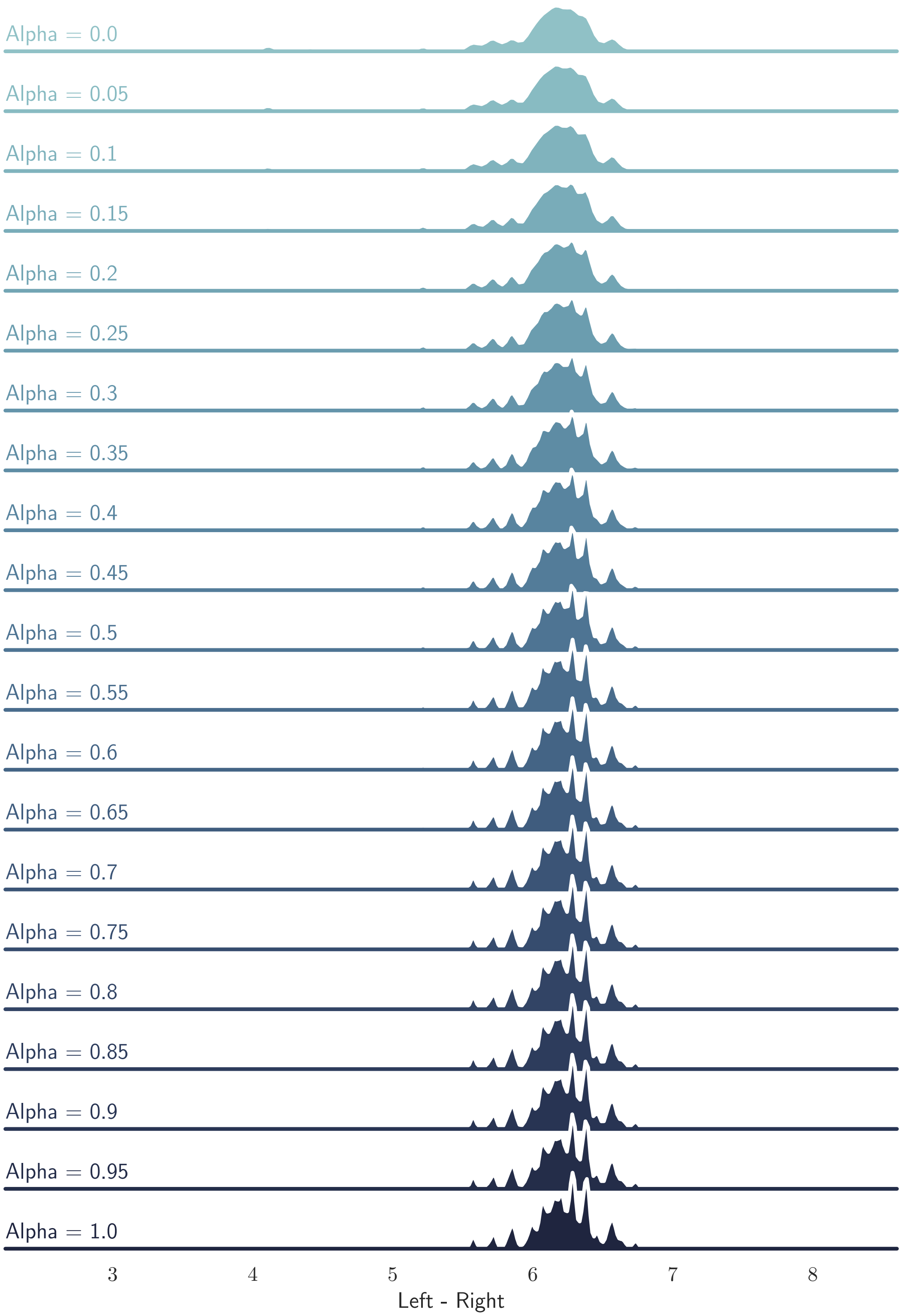

Figure S46. Effect of tweaking political latent dimensions on the political distribution of recommended URLs for far-left (FL) users. We see that extreme media are not recommended anymore in favor of a limited number of mainstream medias (spikes on the distribution).

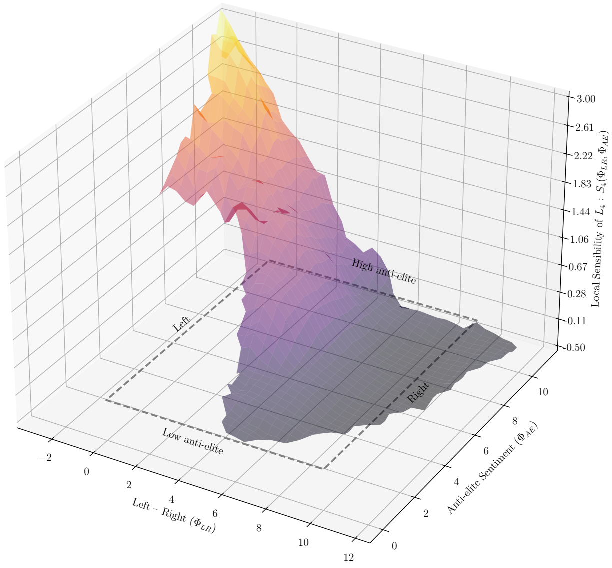

a. Local attribution factor (L_4).
$S^{\text{(Global)}}_{4,\Phi_{LR}} = 0.94$; $S^{\text{(Global)}}_{4,\Phi_{AE}} = 0.29$

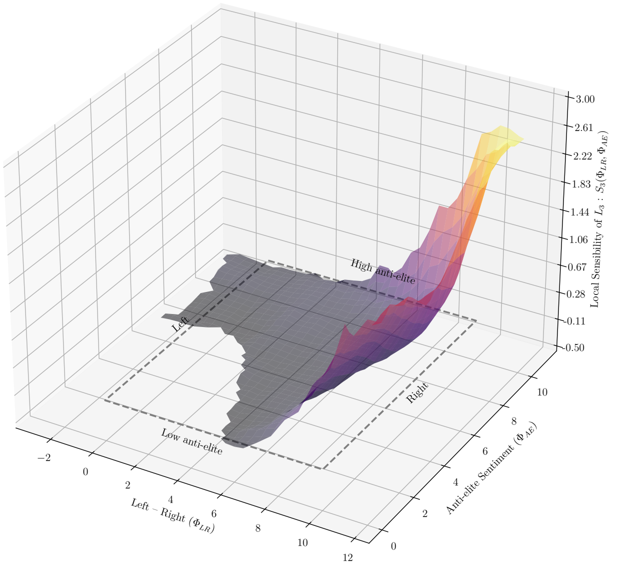

b. Local attribution factor (L_3).
$S^{\text{(Global)}}_{3,\Phi_{LR}} = 0.51$; $S^{\text{(Global)}}_{4,\Phi_{AE}} = 0.10$

Figure S47. The local attribution factor shows the impact of specific political position on the latent dimension of the model. Here we see the local attribution factor of the two dimensions with the highest global attribution factor to Left-Right ideology: L_4 (Left) and L_3 (Right). We see that L_4 reacts to Left and Antie-elite attitudes (a), and that L_3 reacts to Right ideology (b).

Language	Number of users
French	17956
English	2449
Spanish	132
Italian	71
German	62
Arabic	43
Portuguese	28
Catalan	24
Others	181
Unrecognized	1141

Table S1. Main languages used in the description of users

Professional category	Classification CSP2020 *(French)*	Users	Example of occupations
Information, arts and entertainment professions	Professions de l'information, de l'art et des spectacles (3500)	2315	journalist, author, editor, animator, artist, actor, photographer, producer, blogger, film director
Business, IT and administration professionals	Cadres et professions intermédiaires administratives, commerciales et techniques des entreprises (3700, 3800, 4600)	2160	ceo, consultant, entrepreneur, engineer, communications manager, human resources, project manager, coach, analyst, developer, mission manager, designer, accountant, community manager
Elected officers and political representatives	Cadres administratifs et techniques de la fonction publique (3300)	1192	city councilor, mayor, deputy mayor, regional councilor, departmental councilor, member of parliament, parliamentarian, national councilor, parliamentary collaborator, minister, public service
Professors and higher scientific professions	Professeurs et professions scientifiques supérieures (3400)	636	professor, researcher, philosopher, historian, economist, sociologist, psychologist
Legal professions	Professions libérales juridiques et techniques (31B0)	306	attorney, lawyer
Other	Recognized professions in other CSP cathegories	530	physician, farmer, nurse, architect, firefighter, artisan, mediator, hunter

Table S2. Main professional categories identified from users profile description. Occupations related to information, politics and business are highly over represented.

Occupations, Qualifiers and Topics	Interpretation of declared interest
attorney, mediator, member of parliament, national councilor, city councilor, departmental councilor, deputy mayor, regional councilor, mayor, minister, candidate, elected, member, administrator, important member, president, advisor, assistant, city hall, committee, delegate, former, vice-president, general secretary, coordinator, board of directors, support, official account, junior, federation, historian, mission manager, parliamentarian, physician	Politics
researcher, blogger, professor, developer, engineer, enthusiast, alumni, sciencespo, sorbonne, student, association, university, phd, doctor, fan, health, sciences, security, economy, culture, politics, public, finance, history, literature, music, real estate, tourism, diplomacy, research, philosophy, law, sociology, master, lawyer, think tank	Academia
analyst, consultant, architect, artist, designer, ceo, entrepreneur, philosopher, agency, specialist, founder, startup, media, expert, independent, innovation, business, data, development, digital, entrepreneurship, management, marketing, strategy, technology, communication, design, social medias, sustainability, engineering, accountant, thinker	Business
film director, animator, author, journalist, photographer, producer, sociologist, farmer, press	Journalism
actor, activist, citizen, union, agri-food, agriculture, biodiversity and environment, climate, construction, ecology, energy, mobility, justice	Ecology

Table S3. Main groups of co-occurring socio-demographic keywords.

Category	Sub-categories
Mainstream Media	Centre<br>Hyper-centre
Opinion Journalism	Left Wing<br>Right Wing
Periphery	Hyper-local (East)<br>IT & Consumer<br>Leisure and Health<br>PQR & Mag
Counter-Informational Space	Alternative Health<br>Identitarian<br>Revolutionary Right

Table S4. Classification of french medias built from the citation network between medias, proposed by[43]

Code and datasets

The reproducibility code of this paper is available on github.

References

1. Ramaciotti Morales, P. & Cointet, J.-P. Auditing the Effect of Social Network Recommendations on Polarization in Geometrical Ideological Spaces. In *RecSys '21: 15th ACM Conference on Recommender Systems*, DOI: 10.1145/3460231.3478851 (Amsterdam, Netherlands, 2021).
2. Barberá, P. Birds of the Same Feather Tweet Together: Bayesian Ideal Point Estimation Using Twitter Data. *Political Analysis* **23**, 76–91, DOI: 10.1093/pan/mpu011 (2015/ed).
3. Plique, G. *et al.* Ural, a python helper library full of URL-related heuristics. Zenodo, DOI: 10.5281/zenodo.13711837 (2024).
4. Bem, D. J. *Beliefs, Attitudes, and Human Affairs*. Beliefs, Attitudes, and Human Affairs (Brooks/Cole, Oxford, England, 1970).
5. Ajzen, I. *Attitude Structure and Behavior*. Attitude Structure and Function (Psychology Press, 2014).
6. Bond, R. & Messing, S. Quantifying Social Media's Political Space: Estimating Ideology from Publicly Revealed Preferences on Facebook. *American Political Science Review* **109**, 62–78, DOI: 10.1017/S0003055414000525 (2015).
7. Bakker, R., Jolly, S. & Polk, J. Complexity in the European party space: Exploring dimensionality with experts. *European Union Politics* **13**, 219–245, DOI: 10.1177/1465116512436995 (2012).
8. Ramaciotti Morales, P. *et al.* Inferring attitudinal spaces in social networks. *Social Network Analysis Mining* **13**, 14, DOI: 10.1007/s13278-022-01013-4 (2022).
9. Jolly, S. *et al.* Chapel Hill Expert Survey trend file, 1999–2019. *Electoral Studies* **75**, 102420, DOI: 10.1016/j.electstud.2021.102420 (2022).
10. Lowe, W. Understanding Wordscores. *Political Analysis* **16**, 356–371, DOI: 10.1093/pan/mpn004 (2017).
11. Cointet, J.-P. *et al.* De quelle(s) couleur(s) sont les Gilets jaunes ? Plonger des posts Facebook dans un espace idéologique latent. *Statistique et Société* **9**, 79–107 (2021).
12. Ramaciotti Morales, P. *et al.* Atlas Multi-Plateformes d'un Mouvement Social : Le cas des Gilets Jaunes. *Statistique et Société* (2021).
13. Lee, D. D. & Seung, H. S. Learning the parts of objects by non-negative matrix factorization. *Nature* **401**, 788–791, DOI: 10.1038/44565 (1999).
14. Lee, D. & Seung, H. S. Algorithms for Non-negative Matrix Factorization. In *Advances in Neural Information Processing Systems*, vol. 13 (MIT Press, 2000).
15. Mnih, A. & Salakhutdinov, R. R. Probabilistic Matrix Factorization. In *Advances in Neural Information Processing Systems*, vol. 20 (Curran Associates, Inc., 2007).
16. Koren, Y., Bell, R. & Volinsky, C. Matrix Factorization Techniques for Recommender Systems. *Computer* **42**, 30–37, DOI: 10.1109/MC.2009.263 (2009).
17. Hu, Y., Koren, Y. & Volinsky, C. Collaborative Filtering for Implicit Feedback Datasets. In *2008 Eighth IEEE International Conference on Data Mining*, 263–272, DOI: 10.1109/ICDM.2008.22 (2008).
18. Johnson, C. C. Logistic Matrix Factorization for Implicit Feedback Data. *Advances Neural Information Processing Systems* **27**, 9 (2014).
19. Gunawardana, A. & Shani, G. A Survey of Accuracy Evaluation Metrics of Recommendation Tasks. *Journal Machine Learning Research* 2935–2962 (2009).
20. Boutsidis, C. & Gallopoulos, E. SVD based initialization: A head start for nonnegative matrix factorization. *Pattern Recognition* **41**, 1350–1362, DOI: 10.1016/j.patcog.2007.09.010 (2008).
21. Cichocki, A. & Phan, A.-H. Fast Local Algorithms for Large Scale Nonnegative Matrix and Tensor Factorizations. *IEICE Transactions on Fundamentals Electronics, Communications Computer Sciences* **E92.A**, 708–721, DOI: 10.1587/transfun.E92.A.708 (2009).
22. Kennedy, J. & Eberhart, R. Particle swarm optimization. In *Proceedings of ICNN'95 - International Conference on Neural Networks*, vol. 4, 1942–1948 vol.4, DOI: 10.1109/ICNN.1995.488968 (1995).

23. Hu, W. *et al.* Open Graph Benchmark: Datasets for Machine Learning on Graphs, DOI: 10.48550/arXiv.2005.00687 (2021). 2005.00687.

24. Kluver, D., Ekstrand, M. D. & Konstan, J. A. Rating-Based Collaborative Filtering: Algorithms and Evaluation. In Brusilovsky, P. & He, D. (eds.) *Social Information Access: Systems and Technologies*, Lecture Notes in Computer Science, 344–390, DOI: 10.1007/978-3-319-90092-6_10 (Springer International Publishing, Cham, 2018).

25. Yuan, H., Yu, H., Gui, S. & Ji, S. Explainability in Graph Neural Networks: A Taxonomic Survey. *arXiv:2012.15445 [cs]* (2021). 2012.15445.

26. Datta, A., Kovaleva, S., Mardziel, P. & Sen, S. Latent Factor Interpretations for Collaborative Filtering, DOI: 10.48550/arXiv.1711.10816 (2018). 1711.10816.

27. Faverjon, T. & Ramaciotti, P. Discovering ideological structures in representation learning spaces in recommender systems on social media data. In *2023 IEEE/ACM International Conference on Advances in Social Networks Analysis and Mining*, DOI: 10.1145/3625007.3627336 (Kusadasi, Turkey, 2023).

28. Lundberg, S. & Lee, S.-I. A Unified Approach to Interpreting Model Predictions, DOI: 10.48550/arXiv.1705.07874 (2017). 1705.07874.

29. Culotta, A., Ravi, N. K. & Cutler, J. Predicting Twitter User Demographics using Distant Supervision from Website Traffic Data. *Journal Artificial Intelligence Research* **55**, 389–408, DOI: 10.1613/jair.4935 (2016).

30. Ghazouani, D., Lancieri, L., Ounelli, H. & Jebari, C. Assessing socioeconomic status of Twitter users: A survey. In *Proceedings of the International Conference on Recent Advances in Natural Language Processing (RANLP 2019)*, 388–398, DOI: 10.26615/978-954-452-056-4_046 (INCOMA Ltd., Varna, Bulgaria, 2019).

31. Preoţiuc-Pietro, D., Lampos, V. & Aletras, N. An analysis of the user occupational class through Twitter content. In *Proceedings of the 53rd Annual Meeting of the Association for Computational Linguistics and the 7th International Joint Conference on Natural Language Processing (Volume 1: Long Papers)*, 1754–1764, DOI: 10.3115/v1/P15-1169 (Association for Computational Linguistics, Beijing, China, 2015).

32. SOC 2020 - Office for National Statistics. https://www.ons.gov.uk/methodology/classificationsandstandards/standardoccupationalclassif

33. He, Y. & Tsvetkova, M. A Method for Estimating Individual Socioeconomic Status of Twitter Users. *Sociological Methods & Research* 00491241231168665, DOI: 10.1177/00491241231168665 (2023).

34. Sloan, L., Morgan, J., Burnap, P. & Williams, M. Who Tweets? Deriving the Demographic Characteristics of Age, Occupation and Social Class from Twitter User Meta-Data. *PLOS ONE* **10**, e0115545, DOI: 10.1371/journal.pone.0115545 (2015).

35. Wang, Z. *et al.* Demographic Inference and Representative Population Estimates from Multilingual Social Media Data. In *The World Wide Web Conference*, WWW '19, 2056–2067, DOI: 10.1145/3308558.3313684 (Association for Computing Machinery, New York, NY, USA, 2019).

36. Blondel, V. D., Guillaume, J.-L., Lambiotte, R. & Lefebvre, E. Fast unfolding of communities in large networks. *Journal Statistical Mechanics: Theory Experiment* **2008**, P10008, DOI: 10.1088/1742-5468/2008/10/P10008 (2008).

37. Bail, C. *Breaking the Social Media Prism* (Princeton University Press, Tue, 04/06/2021 - 12:00).

38. Başbay, M. M., Elgin, C. & Torul, O. Socio-demographics, political attitudes and informal sector employment: A cross-country analysis. *Economic Systems* **42**, 556–568, DOI: 10.1016/j.ecosys.2018.03.003 (2018).

39. Brown-Iannuzzi, J. L., Lundberg, K. B. & McKee, S. The politics of socioeconomic status: How socioeconomic status may influence political attitudes and engagement. *Current Opinion Psychology* **18**, 11–14, DOI: 10.1016/j.copsyc.2017.06.018 (2017).

40. Kaufmann, K. M. The Gender Gap. *PS: Political Science & Politics* **39**, 447–453, DOI: 10.1017/S1049096506060884 (2006).

41. Présidentielles 2022 : résultats du premier tour par tranche d'âge. https://fr.statista.com/statistiques/1302004/resultats-premier-tour-presidentielles-2022-age/.

42. Piterová, I. & Loziak, A. A Comprehensive Model for Predicting Populist Attitudes. *Journal Social Political Psychology* **12**, 73–88, DOI: 10.5964/jspp.11539 (2024).

43. Cointet, J.-P. *et al.* Uncovering the structure of the French media ecosystem, DOI: 10.48550/arXiv.2107.12073 (2021). 2107.12073.

44. Abbe, E. Community Detection and Stochastic Block Models: Recent Developments. *Journal Machine Learning Research* 86 (2018).
